# Swiss ichthyosaurs: a review

**DOI:** 10.1186/s13358-024-00327-4

**Published:** 2024-09-01

**Authors:** Christian Klug, Timur Sivgin, Feiko Miedema, Beat Scheffold, Achim G. Reisdorf, Iwan Stössel, Erin E. Maxwell, Torsten M. Scheyer

**Affiliations:** 1 Universität Zürich, Paläontologisches Institut, Karl-Schmid-Strasse 4, 8006 Zurich, Switzerland; 2 Staatliches Museum Für Naturkunde Stuttgart, Rosenstein 1, 70191 Stuttgart, Germany; 3 Naturkundemuseum Bamberg, 96047 Bamberg, Germany; 4 Stiftung Ruhr Museum, Fritz-Schupp-Allee 15, 45141 Essen, Germany; 5 Department Erdwissenschaften, ETH Zürich, Sonneggstrasse 5, 8092 Zurich, Switzerland

**Keywords:** Marine reptiles, Exceptional preservation, Konservat Lagerstätten, Taphonomy, Triassic, Jurassic, Cretaceous

## Abstract

Switzerland is an ichthyosaur country: it has a rich record of marine reptile fossils, particularly the fish-shaped ichthyosaurs, and the according research. Here, we provide an overview over the 12 or more genera and at least 13 species plus numerous fragmentary remains of ichthyosaurs from the Triassic to the Cretaceous that have been discovered in twelve cantons thus far, of which four species are based on Swiss holotypes. This wealth of ichthyosaur species can be explained by their abundance in the Middle Triassic conservation deposits (Konservat Lagerstätte) of Monte San Giorgio, as well as occasional discoveries in strata of Middle Triassic to Early Cretaceous age. The moderate abundance of outcrops in reasonable conditions in combination with the long history of palaeontological research in Switzerland explains this good fossil record. In addition to this unique overview, we provide more data for further studies and update the knowledge of these taxa.

## Introduction

The Mesozoic marine revolution coined by Vermeij (1977) does not only concern invertebrates: it could almost be called the great Marine reptile revolution. As summarized by Kelley and Pyenson (2015), at least four important clades of marine reptiles (ichthyosauromorphs, sauropterygians, tanystropheids and thalattosauriforms) originated in the Early Triassic (for the oldest see Huang et al., 2020; Kear et al., 2023). There is no comparable event in the evolution of amniote vertebrates, in that so many disparate clades independently became secondarily aquatic (Kelley & Pyenson, 2015; Motani, 2009), which is not a coincidence; oceanic foodwebs were fundamentally restructured following the greatest marine mass extinction at the Permian–Triassic boundary (e.g. Benton, 2016; Brayard et al., 2017; Chen and Benton, 2012; Fröbisch et al., 2013; Goudemand et al., 2019; Payne & Clapham, 2012; Romano et al., 2013; Scheyer et al., 2014). With the extinction of many groups, ecospace was liberated, but life conditions stayed adverse in wide parts of the world’s oceans. As shown by Brayard et al. (2017) or Flannery-Sutherland et al. (2022), the recovery of marine faunas regionally proceeded faster than in other areas.

The question arises, why did reptiles conquer the seas after having evolved features facilitating life on land? What could have been their advantages compared to marine predators of the Palaeozoic? A seemingly obvious explanation would be that terrestrial vertebrates escaped adverse conditions on land (lack of food) by moving into marine habitats. The absence of increased extinction rates among land plants across the Permian–Triassic boundary (Nowak et al., 2019) casts doubts on this hypothesis; nevertheless, there was also a severe diversity loss among terrestrial vertebrates (e.g., Knoll et al., 2007; Smith & Ward, 2001). Paradoxically, it might have been the fact that the marine reptiles and their ancestors were lung-breathing, which became a selective advantage then. Following the Large Igneous Province-eruptions in Siberia (e.g., Grasby & Bond, 2023), vast parts of the marine realm suffered from low oxygen conditions (e.g., Hülse et al., 2021; Song et al., 2012; Wang et al., 2023). Possibly, the lung-breathing ancestors of the marine reptiles profited from resources in the sea that could not be exploited by the predator groups (e.g., fish) that were already in the sea before the Permian–Triassic boundary extinction. In low-oxygen parts of the sea where low-oxygen-tolerant invertebrates survived while fish could not, lung-breathing forms may have been capable of exploiting these food resources, since they returned to the surface for breathing anyway. This is supported by the abundance of durophagous Ichthyosauromorpha species in the Early Triassic (Huang et al., 2020; Moon and Stubbs, 2020; Økland et al., 2018; Qiao et al., 2022). Answering the question for the ecological framework that enabled the evolution of marine reptiles, however, deserves its own study and in-depth analysis.

At the latest with the renaissance of Mary Anning (e.g., Sharpe, 2020) and her growing global fame, ichthyosaurs have gained new recognition. This was recently further intensified by the discovery of the ‘Rutland dragon’ (Larkin et al., 2023) and some widely recognized articles on giant Late Triassic ichthyosaurs (Sander et al., 2021, 2022). These papers on the huge Triassic forms include another link to Swiss ichthyosaurs: Switzerland has brought forth specimens of both some of the smallest (Middle Triassic *Mixosaurus*, adult body size slightly over a meter in length; Brinkmann, 1996; Huene, 1939) and remains of some of the largest known ichthyosaurs (Late Triassic Shastasauridae which, as reconstructed, would reach and potentially exceeded 20 m in length; see Sander et al., 2021, 2022).

Maisch et al. (2008) gave a first cursory overview of ichthyosaur material from Switzerland. To our knowledge, the history of ichthyosaur discoveries in Switzerland and the according research started with the finding of isolated ichthyosaur vertebrae in Laufenburg AG (for canton abbreviations see Methods-chapter). These were first mentioned by Moesch (1857) and later described in detail by von Huene (1916). Wiman (1912) discussed and figured *Mixosaurus cornalianus* from Cava Trefontane. Still in the nineteenth century, the famous skeleton of *Stenopterygius* was excavated from the ‘Posidonienschiefer’ (Toarcian) of Teysachaux in 1870 (Ooster & Fischer-Ooster 1870–1871; Weidmann, 1981). It was published by von Huene (1939), i.e. when the first ichthyosaurs had already been discovered in Ticino. By then, the first ichthyosaurs from the Italian side of Monte San Giorgio had already been described by Bassani (1886) and Peyer commenced excavations in the Ladinian Grenzbitumenzone on the Swiss side in 1924. With the beginning of Peyer’s research endeavours, a first wave of publications on Swiss ichthyosaurs appeared in the 1930s by Peyer and von Huene, followed by a long interval with only few publications.

The oldest entries of *Mixosaurus* in the collection of the Department of Palaeontology at the university of Zurich date back to 1925, i.e. one year after the beginning of Peyer’s excavations (see Sues, 2024). This highlights the great abundance of mixosaurid remains in Monte San Giorgio. In the 1930s, Peyer and Koechlin (1934) published on ichthyosaurs from northern Switzerland such as the ‘Bornsaurier’, a disarticulated platypterygiine colloquially named after the mountain Born (near Ruppoldingen SO), and one from Grellingen BL (Peyer & Koechlin 1934). During World War II, nothing was published on ichthyosaurs in Switzerland to our knowledge. After the war, Besmer (1947) was likely the first to resume ichthyosaur research in Switzerland. Much later, Früh (1962) published on Jurassic ichthyosaurs from Beggingen SH. The next three decades were rather quiet; a second wave began in the late 1980s and through the 1990s by Furrer and Brinkmann, followed by several papers by Maisch between 1997 and 2014 and the most recent one by Miedema et al. (2024). This phase began with the material from Plan Falcon (Yvorne) VD, which was published by Mettraux and Mohr (1989). In the same year, *Cymbospondylus buchseri* was introduced by Sander (1989). Important discoveries of mostly disarticulated shastasaurid material in the canton Grison were made by Furrer (1993) during field work for his dissertation. This material recently received more attention in a review of European shastasaurids from the Late Triassic (Sander et al., 2022). In the late 1990s to the early 2000s, Brinkmann (1996, 1997, 1998a, b, 1999, 2001, 2004) wrote a series of monographs and shorter articles about Triassic ichthyosaurs. Around the same time, Cook (1994) provided a preliminary description of material from Monte San Giorgio. Starting in the late 1990s, numerous post-Triassic specimens were studied. Maisch and colleagues published over ten articles featuring Swiss ichthyosaurs (Maisch, 2014; Maisch & Matzke, 1997, 1998, 2000, 2005; Maisch & Reisdorf, 2006a, b; Maisch et al., 2008; Reisdorf, 2007; Reisdorf et al., 2011, 2012). Their materials mostly came from northern Switzerland but also from Ticino. In the same interval, Ayer (2003) documented the ichthyosaurs from La Presta NE while den Brok et al. (2004) focused on materials from canton Basel Landschaft (Buckten BL and Ormalingen BL). Focusing on sedimentology and ichnology, Wetzel & Reisdorf (2007) analysed the host strata of the *Hauffiopteryx* from Unterer Hauenstein SO. Kolb et al. (2011) used Monte San Giorgio specimens of *Mixosaurus* to study their bone histology. Most recently, Maxwell (2012) looked at intraspecific variation in phalangeal counts and limb structure in *Mixosaurus cornalianus*, Pardo-Pérez et al. (2020) found pathologies in mixosaurids and other ichthyosaurs, and Bindellini et al. (2021) re-examined *Besanosaurus*, Sander et al. (2022) revised the giant shastasaurids from eastern Switzerland that had been discovered by Furrer, Miedema et al., (2023a, b) studied the ontogeny and reproductive biology of *Mixosaurus* and a new early ophthalmosaurian was described by Miedema et al. (2024).

Following this condensed overview of the history of palaeontological research on Swiss ichthyosaurs, we shortly portray most of ichthyosaur remains known from Switzerland. It is neither possible nor our aim to provide an exhaustive list of specimens (i.e., listing every bone fragment that could be ichthyosaurian in every collection) because new materials are discovered every year. Instead, we aim at offering a good overview over the taxa known from Switzerland, providing state-of-the-art illustrations of the main specimens, and including the main publications.

## Abbreviations

The respective cantons are abbreviated in many places using the official two-letter abbreviations: AG—Aargau; AR—Appenzell Ausserrhoden; BE—Bern; BL—Basel Landschaft; FG—Fribourg; JU—Jura; NE—Neuchatel; SG—St. Gallen; SO—Solothurn; SH—Schaffhausen; VD—Vaud; VS—Vallais

Institutional abbreviations: BES SC—Museo di Storia Naturale di Milano, Milan, Italy. GPIT—Paläontologische Sammlung der Universität Tübingen, Tübingen, Germany. MGL—Musée Géologique de Lausanne, Lausanne, Switzerland. Nat—Museum zu Allerheiligen, Schaffhausen, Schaffhausen, Switzerland. NMB—Naturhistorisches Museum Basel, Basel, Switzerland. NMBE—Naturhistorisches Museum Bern, Bern, Switzerland. NMO—Naturmuseum Olten, Olten, Switzerland. NMSG—Naturmuseum St. Gallen, St. Gallen, Switzerland. PIMUZ—collections of the Department of Paleontology of the University of Zurich, Zurich, Switzerland. SMF—Sauriermuseum Frick, Frick, Switzerland. PK—Collection Peter Kürsteiner at the Naturmuseum St. Gallen, Switzerland. REG—Muséum d’histoire naturelle de Neuchâtel, Neuchâtel, Switzerland.

## Results

Our survey is partially based and expanding on the overview provided by Maisch et al. (2008). To complete the picture, we contacted curators at some museums in the big cities in Switzerland. Examples of Swiss ichthyosaur collections in Bern, Basel and Lausanne are listed in Tables 1, 2, and 3. Please note that there are more specimens, often rather fragmentary or consisting of isolated bones, in many smaller museums, which we could not include in this review. The collection in Zurich comprises several hundred specimens, often complete, articulated or semi-articulated (see also Beardmore & Furrer, 2016), so we refrained from preparing a table and refer the reader to the online portal of the database: https://www.pim.uzh.ch/apps/cms/pageframes/sammlung_db.php

**Table 1:** Ichthyosaur remains in the Naturhistorisches Museum Bern (provided by Bernhard Hostettler)

Object	Coll Nr	Lithostrat	Biostrat	Locality
4 vertebrae	FPJ 3307	Staffelegg Fm. Gross-Wolf Mb	Insignis-Zone	claypit Fasiswald
1 humerus	FPJ 25602	Staffelegg Fm. Gross-Wolf Mb., Erlimoos-Bed	Variabilis-Zone	claypit Fasiswald
1 upper jaw?	FPJ25609	Staffelegg Fm. Gross-Wolf Mb., Erlimoos-Bed	Variabilis-Zone	claypit Fasiswald
1 jaw ramus	FPJ 25608	Staffelegg Fm. Gross-Wolf Mb., Erlimoos-Bed	Variabilis-Zone	claypit Fasiswald
1 large vertebra	FPJ 25625	Staffelegg Fm. Gross-Wolf Mb., Erlimoos-Bed	Variabilis-Zone	claypit Fasiswald
3 large phalanges	FPJ 21526, 25599, 25601	Staffelegg Fm. Gross-Wolf Mb., Erlimoos-Bed	Variabilis-Zone	claypit Fasiswald
1 femur?	25597	Staffelegg Fm. Gross-Wolf Mb., Erlimoos-Bed	Variabilis-Zone	claypit Fasiswald
diverse vertebrae	FPJ25624, 25623, 25621, 25620, 21519–21524, 21525	Staffelegg Fm. Gross-Wolf Mb., Erlimoos-Bed	Variabilis-Zone	claypit Fasiswald
Diverse unidentifiable bone fragments	Coll FPJ	Staffelegg Fm. Gross-Wolf Mb., Erlimoos-Bed	Variabilis-Zone	claypit Fasiswald
1 vertebra	NMBE D2694	Lias		Densbüren
1 vertebra	FPJ 19842	Staffelegg Fm. Gross-Wolf Mb., Eriwies-Bed	Aalensis-Zone, Torulosum-Subzone	claypit between Bretzwil & Seewen
1 vertebra	FPJ 1019	Passwang Fm., Sissach Mb	Aalenian	
1 vertebra	NMBE Coll A. Romano 511	Burghorn Fm., Wettingen Mb		Born near Olten
1 vertebra	FPJ 3277	Reuchenette Fm., Banné Mb	Cymodoce/Acanthicum-Zone, Kimmeridgian	Construction site
1 vertebra	NMBE Coll A. Romano 509	Reuchenette Fm., Banné Mb	Cymodoce/Acanthicum-Zone Kimmeridgian	Vendlincourt, quarry W Corchevez

**Table 2:** Ichthyosaur remains in the Naturmuseum Basel (provided by Loïc Costeur)

Taxon	Object	Coll. Nr	Stage	Locality	Donator
*Phalarodon* sp. (*P. atavus*?)	distal upper/lower jaw	S.Tr.12	Anisian	Schwaderloch AG	Dr. Vosseler, 1918
Ichthyosauria indet	vertebrae fragments	S.Tr.13–14	Anisian	Schwaderloch AG	Dr. Vosseler, 1918
*Mixosaurus* sp.	diverse fragments	F.O.7, 21, 22, 23, 24, 25, 26, 32, 42	Ladinian	Tre Fontane TI	
Ichthyosauria indet. ?	2 vertebrae	7390–7391	Hettangian	Frick AG	F. Woltersdorf, 1915
aff. *Temnodontosaurus* sp.	2 skull fragments (jaw)	L.D.35, 36	Sinemurian	Canton Basel	
Ichthyosauria indet	1 vertebra	L.D.39	Sinemurian	Hallau SH	Th. Engelmann, 1920
Ichthyosauria indet	tooth	L.D.31	Sinemurian?	Canton Basel	F. Seul
Ichthyosauria indet	1 vertebra	L.D.26	Middle Liassic	Wartenberg BL?	
Ichthyosauria indet	ca. 20 fragments	L.D.38	Aalenian	Buckten BL	Prof. Abl Müller, 1859
Ophthalmosauria indet	tooth fragment	No Nr	Callovian	Herznach AG	F. Woltersdorf, 1948
Ichthyosauria indet	1 vertebra	M.H.447	Oxfordian	Kastelental near Grellingen BE	E. Koechlin, 1931
Ichthyosauria indet	1 vertebra	No Nr		Coin du Bois near Porrentruy JU

**Table 3:** Ichthyosaur remains in the Museum cantonal des sciences Lausanne (provided by Antoine Pictet)

Taxon	Object	Coll. Nr	Stage	Locality	References
*Mixosaurus* sp.	thorax	39548	Anisian	Monte San Giorgio TI	
Ichthyosauria indet	slab with ribs	48134	Rhaetian	Ruisseau du Chalevey, Montreux VD	Furrer (1960)
Ichthyosauria indet	several bones	48135	Rhaetian	Plan Falcon VD	
Ichthyosauria indet	tooth fragment	48137	Rhaetian	Plan Falcon VD	
Ichthyosauria indet	bone	48138	Rhaetian	Plan Falcon VD	
Ichthyosauria indet	bone	48139	Rhaetian	Plan Falcon VD	
Ichthyosauria indet	fin	40198	Toarcian	Termen quarry, Brig VS	
?*Stenopterygius* sp.	fragments and vertebra	40947	Toarcian	Ruisseau Chalevay, Montreux VD	Maisch and Reisdorf (2006a, b); Weidmann (1981)
Ichthyosauria indet	vertebra	40948	Toarcian	Ruisseau Chalevay, Montreux VD	Maisch and Reisdorf (2006a, b)
Ichthyosauria indet	vertebra	40949	Toarcian	Ruisseau Chalevay, Montreux VD	Maisch and Reisdorf (2006a, b)
Ichthyosauria indet	rib	40950	Toarcian	Ruisseau Chalevay, Montreux VD	Maisch and Reisdorf (2006a, b); Weidmann (1981)
?*Stenopterygius* sp.	rib and other fragments	42001	Toarcian	Ruisseau du Chalevey, Montreux VD	Maisch and Reisdorf (2006a, b); Weidmann (1981)
?*Stenopterygius* sp.	slab with ribs	42002	Toarcian	Ruisseau du Chalevey, Montreux VD	Maisch and Reisdorf (2006a, b); Weidmann (1981)
?*Stenopterygius* sp.	slab with ribs	42003	Toarcian	Ruisseau du Chalevey, Montreux VD	Maisch and Reisdorf (2006a, b); Weidmann (1981)
?*Stenopterygius* sp.	slab with ribs	42004	Toarcian	Ruisseau du Chalevey, Montreux VD	Maisch and Reisdorf (2006a, b); Weidmann (1981)
Ophthalmosauria?		3785	Bathonian	Arête des Verraux below Coursy FR	
Ophthalmosauria	fragments & vertebra	40204	Hauterivian	Forêt de Pateroux, right bank of Gorges de l'Orbe VD	

*Stratigraphic range:* Our literature research combined with research in museum collections shows that, in Switzerland, ichthyosaur remains are distributed from the Anisian (c. 242 Ma, both from Monte San Giorgio and northern Switzerland) to the Aptian (c. 120 Ma, Alpstein and La Presta); thus, these remains cover about 122 million years of ichthyosaur evolution. Currently, ichthyosaurs are known from the Olenekian of Svalbard (251 Ma, Kear et al., 2023) to the late Cenomanian (93 Ma; Acikkol, 2015; Bardet, 1992; Fischer et al., 2016). Fischer et al. (2016) list a total stratigraphic range of ichthyosaurs of 157 million years, i.e. we are missing only 35 million years of ichthyosaur evolution, mainly in the Cretaceous (27 out of 35 Ma), where diversity was declining anyway. This is somewhat surprising since outcrops of Albian and Cenomanian marine sediments are quite widespread, although mostly in alpine regions.

**Fig. 1:**
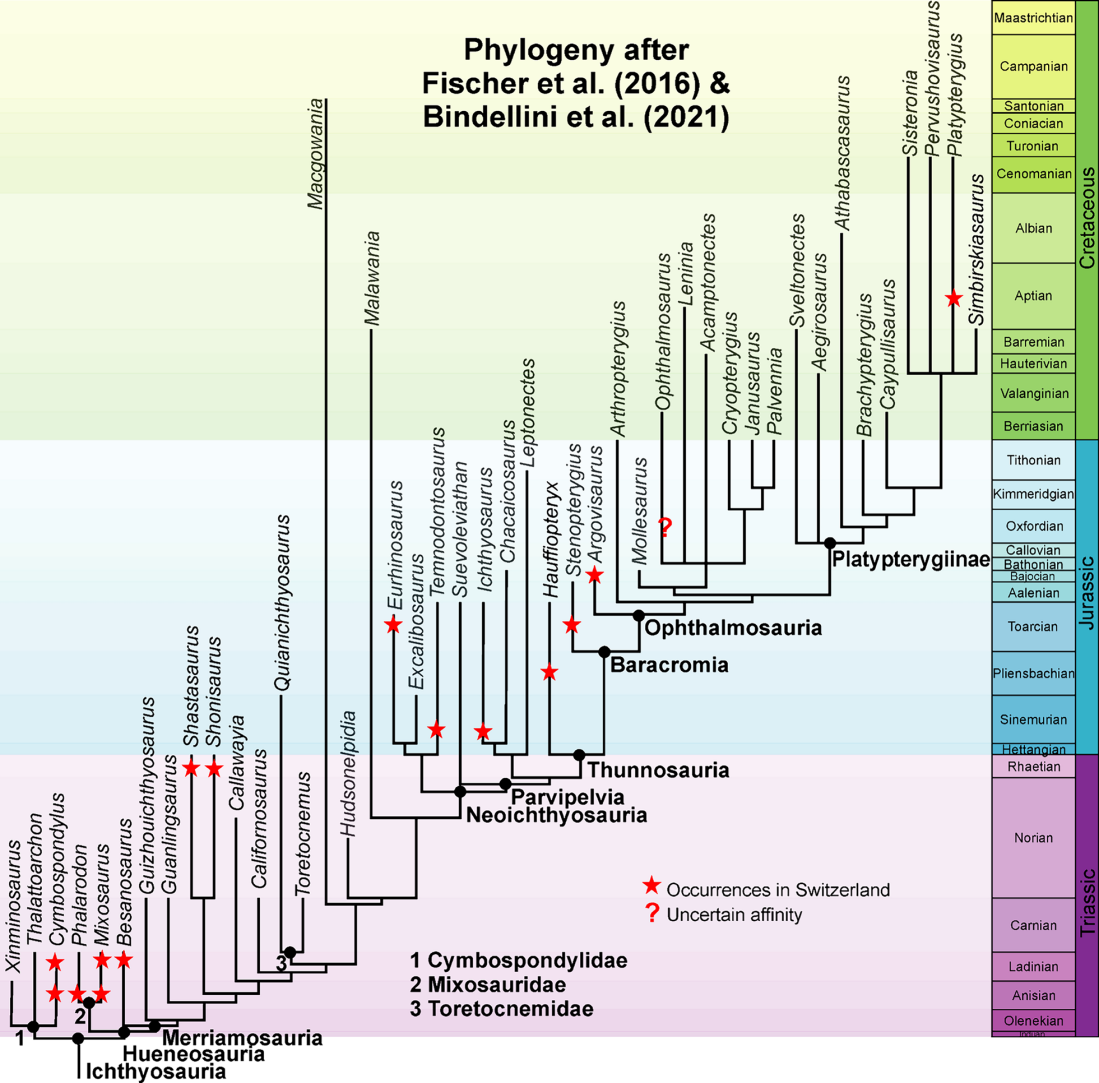
Phylogeny of ichthyosaurs with the positions of taxa occurring in Switzerland. Triassic phylogeny after Bindellini et al. (2021) and Jurassic as well as Cretaceous phylogeny after Fischer et al. (2016)

*Chronostratigraphical distribution:* We demonstrate here that Swiss occurrences of ichthyosaurs cover almost the full known range of the clade (Fig. 1). Like occurrences in other countries (Cleary et al., 2015), the greatest abundances and diversity are found in the Middle Triassic (Anisian) to Early Jurassic (Toarcian). Post-Toarcian ichthyosaur remains are quite rare in Switzerland and only the genus *Argovisaurus* (Miedema et al., 2024) and a platypterygiine («Bornsaurier»; Maisch, 2014) are represented by more complete materials. Isolated bones occur occasionally in sediments of Middle to Late Jurassic age, but in the Cretaceous, they become extremely rare.

Particularly the early branching forms like *Mixosaurus* and *Cymbospondylus* are quite well represented in the Middle Triassic. Shastasaurids (Bindellini et al., 2021; Sander et al., 2022) are not common but remains have been published from several localities. In the Early Jurassic deposits, the abundance of ichthyosaur remains varies. They have been documented in numerous strata from the Hettangian to the Toarcian, although mostly as isolated elements (see Tables 1, 2, 3).

**Fig. 2:**
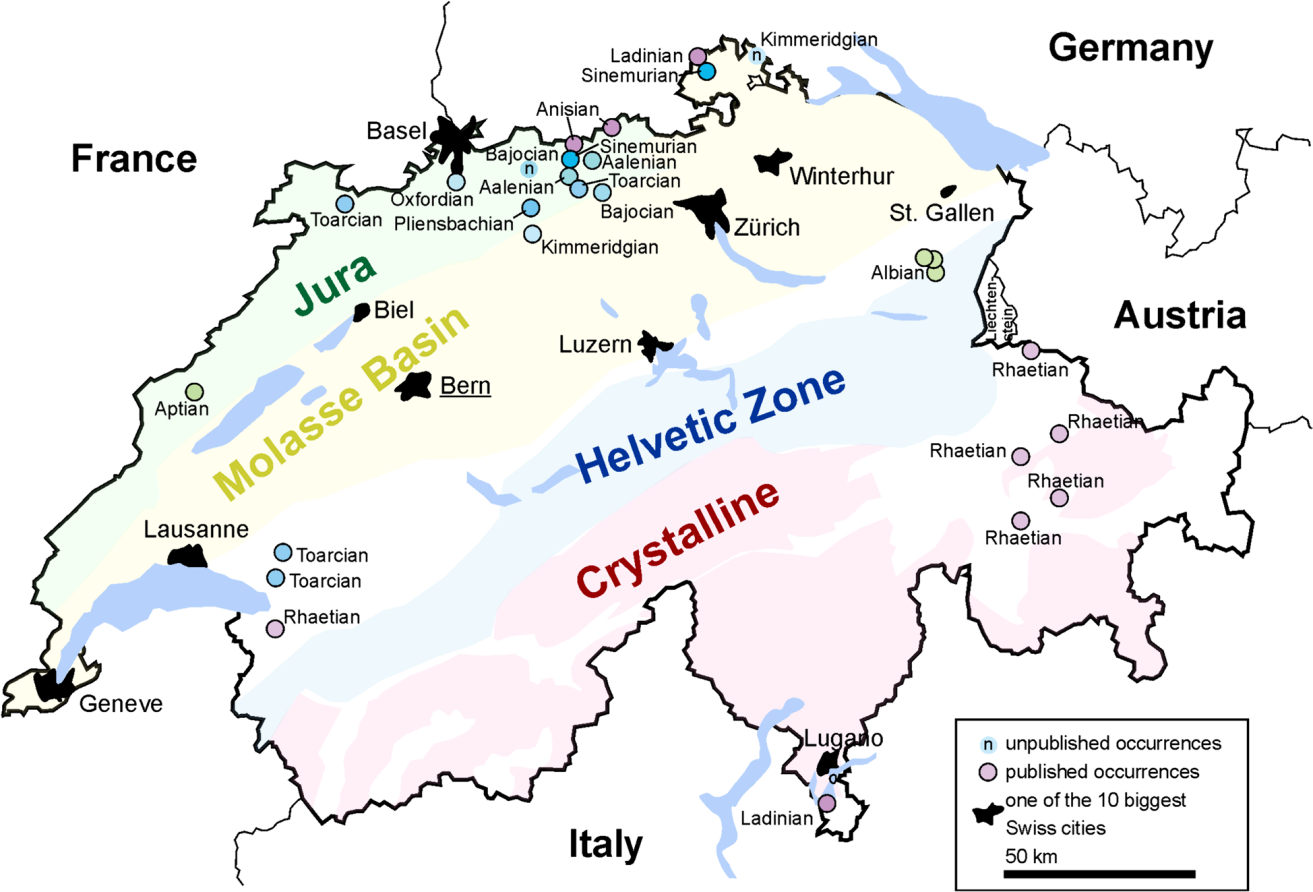
Map of Switzerland with all occurrences currently published or known to us but not studied yet. The four main geologic units of Switzerland are shown, modified after a map from Nagra.ch (the national association for the storage of radioactive waste): light green: Folded and Tabular Jura in the N and NW; yellow: Swiss Plateau and Molasse Basin; light blue: Northern Alps with the Helvetic Zone; pink: Central and Southern Alps with the Crystalline Zone. Some of the occurrences were taken from Maisch et al. (2008)

*Geographic distribution:* The geographic distribution of ichthyosaurs was already depicted by Maisch et al., (2008: fig. 1). We added some additional occurrences. In Fig. 2, we show both the main geological units and the published occurrences of ichthyosaurs.

Mesozoic sediments in Switzerland occur in the northern and western part of Switzerland (Jura Mountains) as well as in the northern and southern part of the Swiss Alps (e.g., Weissert & Stössel, 2015; the following information is from this source). On the one hand, they document the break-up of the continents, the opening of an ocean, the subduction of this ocean, the renewed collision of continents and the formation of the alpine orogen. On the other hand, they cover a wide spectrum of depositional environments: they range from shallow epicontinental seas on the Eurasian continent, the transition to deep marine sediments of the Tethyan Piemont-Ligurian Ocean, including the shallower Briançonnais-Microcontinent, and finally the shallow marine seas of the continental margin of Adria, the promontory of the African continent (e.g., Funk et al., 1993). All these sediments were deformed to varying degrees and incorporated into the alpine mountain belt.

Jurassic occurrences are mostly documented from the Swiss Jura mountains but also from the canton Vaud, surrounding the few Cretaceous records from the Alpstein and La Presta (Neuchâtel). Logically, the crystalline part of the Swiss Alps lacks ichthyosaurs records entirely. In contrast to the French and British parts of the margin of the Eurasian continent (Fischer et al., 2014), the Swiss Helvetic Zone is surprisingly poor in records. The scarcity of ichthyosaurs can be explained best by the low diversity of Cretaceous ichthyosaurs in general and by the mostly quite shallow marine facies (carbonate platforms) of Early Cretaceous to Cenomanian-aged rocks in what is today Switzerland (Föllmi, 1989).

## Systematic palaeontology

We ordered the Swiss specimens stratigraphically and within the periods systematically. The systematic parts and thus the taxonomy were largely taken from Bindellini et al. (2021) and Fischer et al. (2016). Taxonomic identification further followed the referenced literature. Non-diagnostic specimens are listed at the end of this section. Please note that the short description sections added below are not to be confused with official species (comparative) description paragraphs.

### Triassic

Ichthyosauria Blainville, 1835

Hueneosauria Maisch & Matzke, 2000

Incertae sedis

*Wimanius odontopalatus* Maisch & Matzke, 1998

Figure 3

Material: Holotype GPIT-PV-76272.

Locality: Monte San Giorgio (Ticino)

Stratigraphic position: Besano Formation, Middle Triassic, Anisian.

Short description: Only the skull is known (Maisch & Matzke, 1998). The tooth-bearing part is completely preserved while the posterior part of the skull is missing some bones (for details see Maisch & Matzke, 1998). The rostrum is long and slender, with moderately stout conical teeth. Based on the skull length, the animal was probably close to *Mixosaurus cornalianus* in size (c. 1.5 m in length).

Remarks: *Wimanius* was described by Maisch and Matzke (1998) based on a skull preserved in ventral view in the collection of Tübingen. They argued for a new taxon based on the “isodontous and thecodontous dentition” (p. 38). The species was considered as incertae sedis (Motani, 1999), a potential indeterminate shastasaurid (Sander, 2000) or a synonym of *Besanosaurus leptorhynchus* (McGowan & Motani, 2003). *Wimanius* was not explicitly discussed in a recent re-evaluation of the latter species (Bindellini et al., 2021). *Wimanius* is sometimes regarded as having mixosaurid affinities (e.g., Maisch & Matzke, 2000), although a more derived position has also been proposed (Moon, 2019). *Wimanius* is here provisionally regarded as an additional valid ichthyosaur taxon present at the Monte San Giorgio locality pending re-evaluation.

**Fig. 3:**
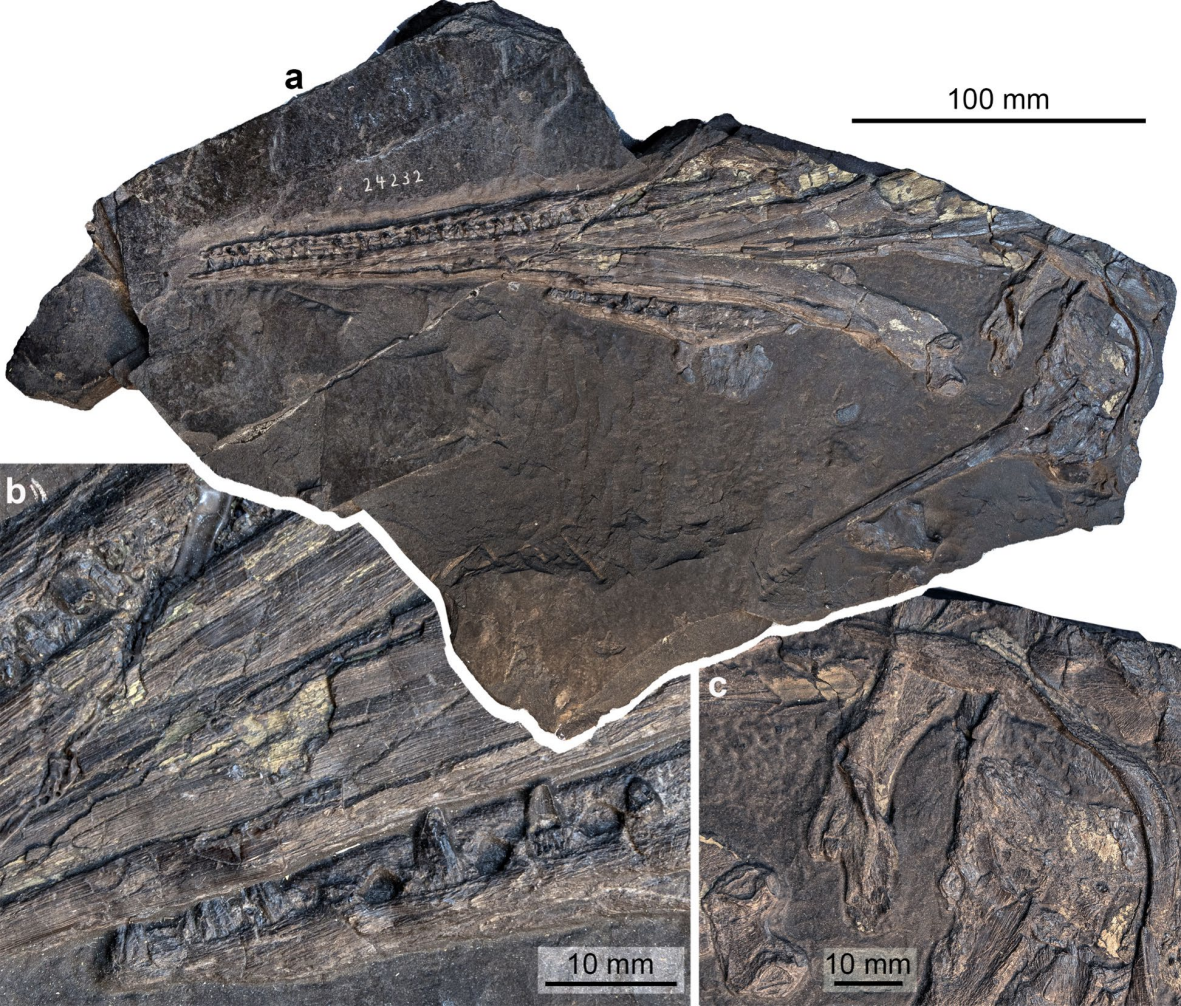
*Wimanius odontopalatus* Maisch & Matzke, 1998, holotype, GPIT-PV-76272. Photos courtesy of G. Bindellini (Milano). **a** The complete holotype. **b** Detail showing a part of the dentition. **c** Disarticulated bones from the posterior part of the skull

Mixosauria Baur, 1887

Mixosauridae Baur, 1887

*Mixosaurus cornalianus* (Bassani, 1886)

 Figures 4, 5

Material: Neotype PIMUZ T 2420 (Brinkmann, 1999) and many other materials such as, e.g., PIMUZ T4857.

Locality: Monte San Giorgio (Ticino)

Stratigraphic position: Besano Formation (also formerly referred to as Grenzbitumenzone) Middle Triassic, Anisian

Short description: The neotype is an adult specimen (sensu Miedema et al., 2023a), almost complete and preserved in right ventrolateral view. However, *M. cornalianus* is a small and quite abundant species (> 300 specimens in the collections of the Department of Palaeontology, University of Zurich), usually less than 1.5 m long (Sander et al., 2021). It has a slender rostrum, the big orbits characteristic of many ichthyosaurs, a rather deep body and a caudal axial skeleton with only a very slight bend. The teeth were rather small (Brinkmann, 2004). *Mixosaurus* had longer anterior than posterior limbs with five digits (Brinkmann, 2004). Renesto et al. (2020) demonstrated the presence of a dorsal fin in *Mixosaurus cornalianus*. The caudal fin was mainly a dorsal connective tissue plate, unlike in post-Triassic forms.

Remarks: Live birth was not always head or tail first (Brinkmann, 1996). In any case, the sample-size of in situ embryos is very low (Miedema et al., 2023a, b: p. 5). Preserved gastric contents include cephalopod hooklets (Brinkmann, 1997), and in one case scales of small fishes (Renesto et al., 2020).

**Fig. 4:**
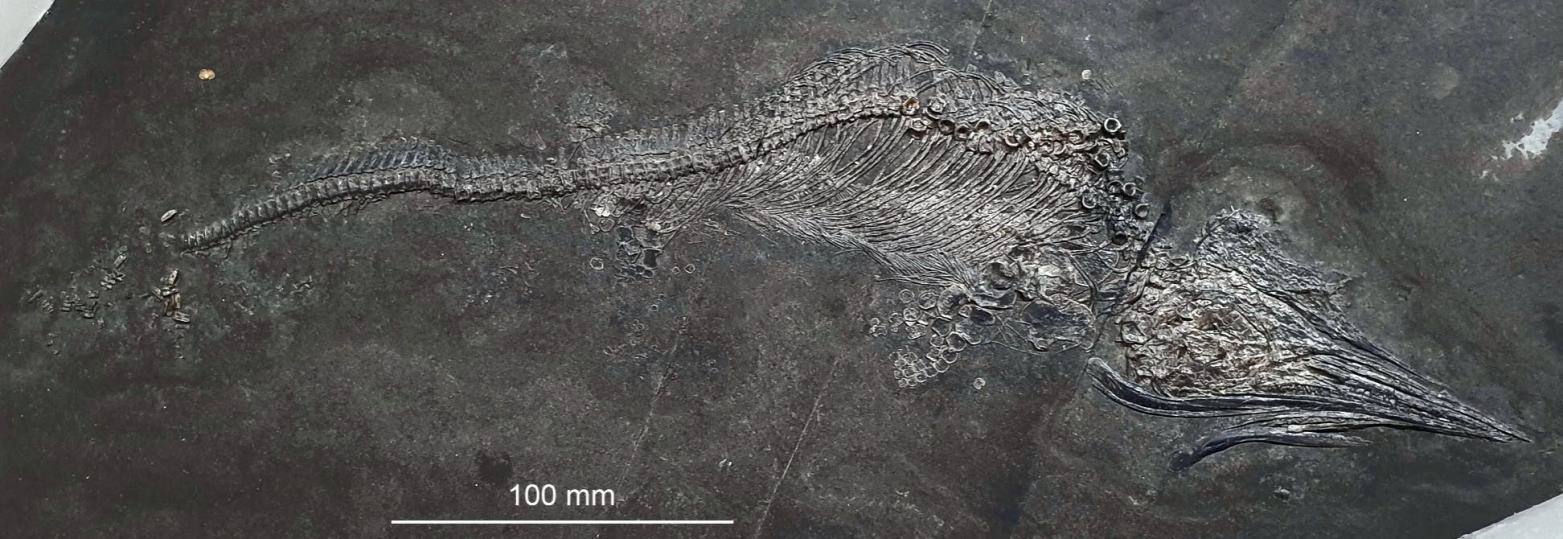
*Mixosaurus cornalianus* (Bassani, 1886), PIMUZ T4857, likely juvenile individual on display in the Natural History Museum of the University of Zurich

*Mixosaurus kuhnschnyderi* (Brinkmann, 1998b) 

Figure 6

**Fig. 5:**
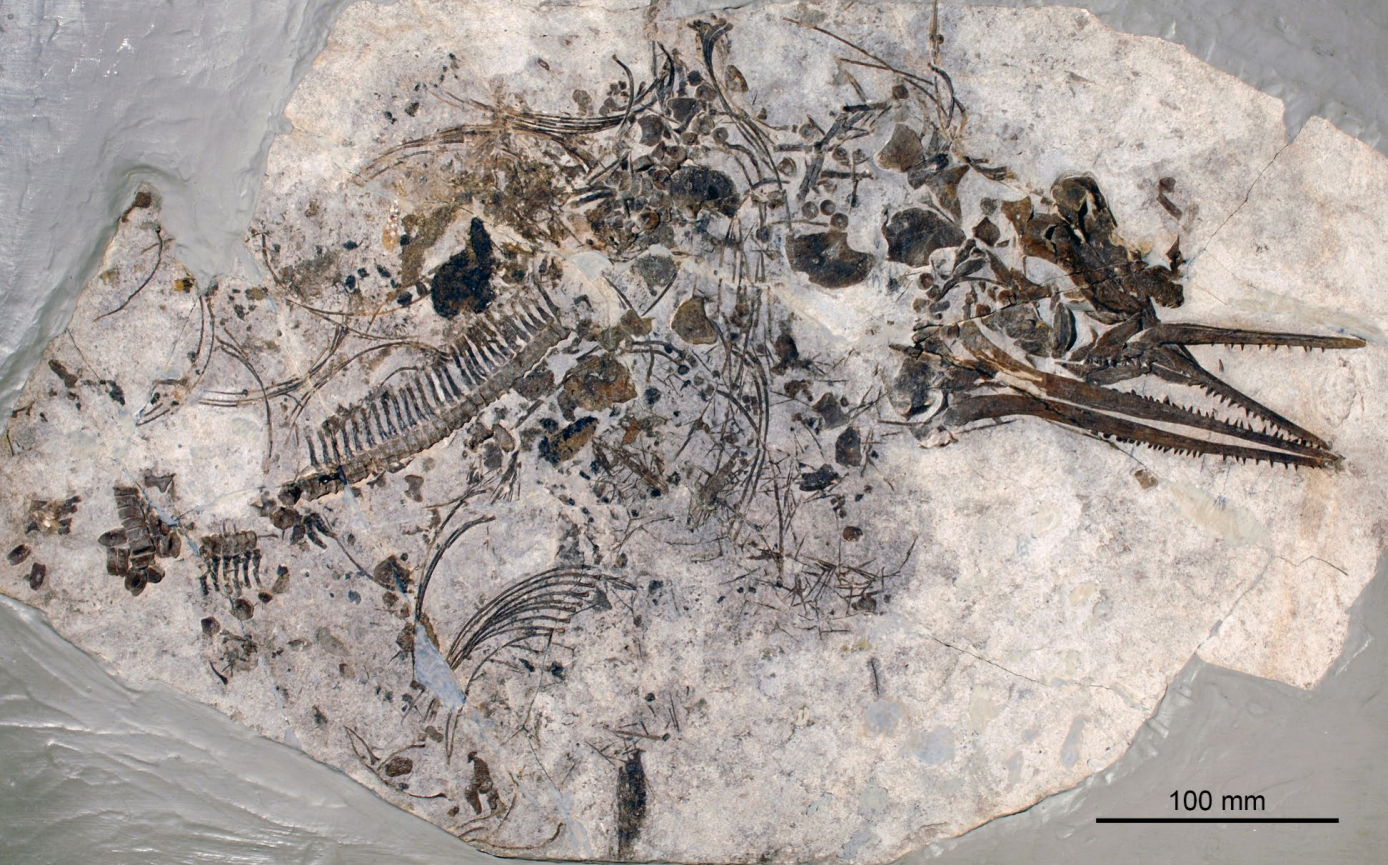
*Mixosaurus kuhnschnyderi* (Brinkmann, 1998a, b), PIMUZ T1324, holotype, Punkt 902, Monte San Giorgio; immature, incomplete, and slightly disarticulated individual

Material: Holotype PIMUZ T 1324 (Fig. 5) and a referred specimen housed in Milan (Brinkmann, 1998a).

Locality: Monte San Giorgio (Tessin, also Italy).

Stratigraphic position: Besano Formation, Middle Triassic, Anisian.

Short description: Two specimens of *M. kuhnschnyderi* are known, the osteologically immature (sensu Miedema et al., 2023a) and extensively disarticulated holotype, and a larger referred specimen, consisting of an articulated skull preserved and fragmentary postcranium in lateral view (Brinkmann, 1998a). *M. kuhnschnyderi* is a small ichthyosaur, less than 1.5 m long. The skull is similar to *M. cornalianus*, but conical and rounded crushing teeth alternated in the posterior part of the jaw (Brinkmann, 1998a, b; McGowan & Motani, 2003).

*Remarks*: The species was formerly included in the genus *Sangiorgiosaurus* Brinkmann, 1998b (McGowan & Motani, 2003: p. 69).

**Fig. 6:**
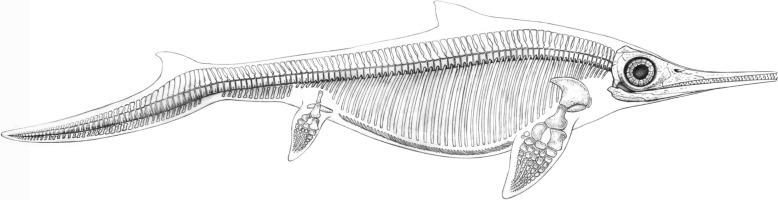
*Mixosaurus cornalianus* (Bassani, 1886), Middle Triassic; skeletal reconstruction by Beat Scheffold

*Phalarodon* sp. 

Figure 7

**Fig. 7:**
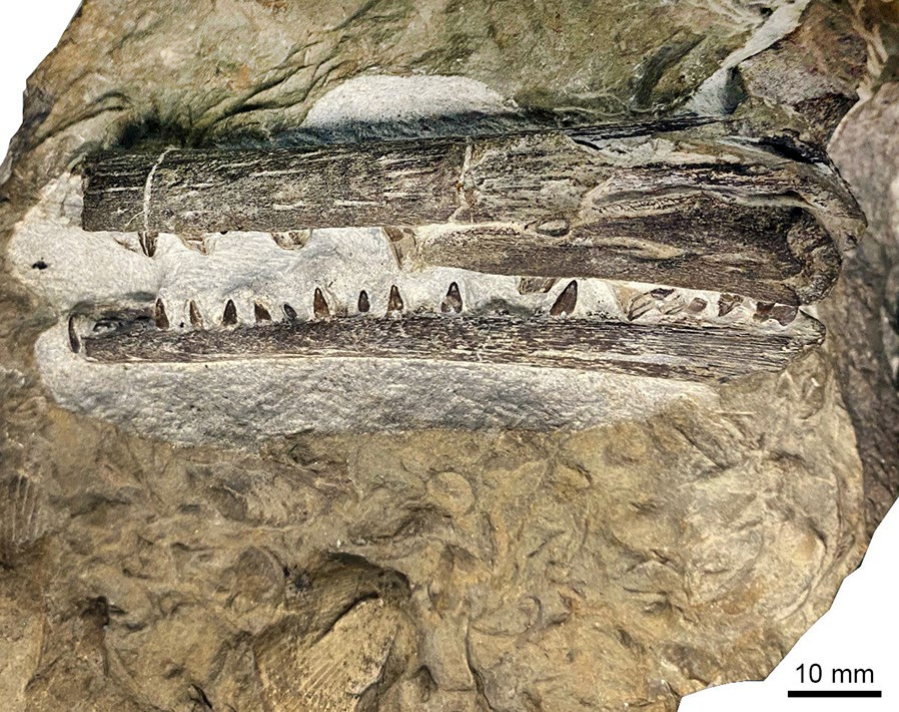
*Phalarodon* sp. (von Huene, 1916), NMB S. Tr. 12, Wellenkalk of Schwaderloch AG; skull fragment preserving parts of the dentary (bottom), premaxilla, maxilla, vomer, and quadrate (see Maisch & Matzke, 2005: Fig. 1). The slab also bears the bivalves *Plagiostoma lineata* (ribbed) and *Hoernesia socialis*. Photo modified after Loïc Costeur (Basel)

Material: PIMUZ T1311, NMB S. Tr. 12 (Fig. 7).

Locality: Monte San Giorgio TI, Laufenburg AG

Stratigraphic position: Besano Formation, and Lower Muschelkalk, Anisian, Middle Triassic

Short description: PIMUZ T1311 is almost complete and articulated, although the skull is somewhat disrupted, whereas NMB S. Tr. 12 displays the incomplete left premaxilla, maxilla, vomer, quadrate and dentary. NMB S. Tr. 12 lacks the supra- and subnarial processes of the premaxilla, supporting referral to Mixosauridae (Roberts et al., 2022). Characteristically for the genus *Phalarodon* (Roberts et al., 2022), both NMB S. Tr. 12 and PIMUZ T1311 show a mandibular heterodonty with posterior teeth being larger, and stouter with thecodont implantation.

Remarks: The fragmentary skull from Aargau (NMB S. Tr. 12) was published by Maisch and Matzke (2005) and assigned to *Phalarodon major*. McGowan and Motani (2003) considered *P. major* to be a nomen dubium based on a non-diagnostic lectotype. *Phalarodon* currently has three valid species, *P. atavus* (revision by Liu et al., 2013), *P. fraasi*, and *P. callawayi*, with only *P. atavus* documented from the Muschelkalk Group. The Laufenburg skull is here referred to *Phalarodon* sp. pending further revision of the Lower Muschelkalk Group forms. Brinkmann (2004) described a small specimen from Monte San Giorgio that was referred to *Phalarodon* sp. (PIMUZ T1311).

Hueneosauria Maisch & Matzke, 2000

Cymbospondylidae Huene, 1948

*Cymbospondylus buchseri* Sander, 1989

Figures 8, 9

**Fig. 8:**
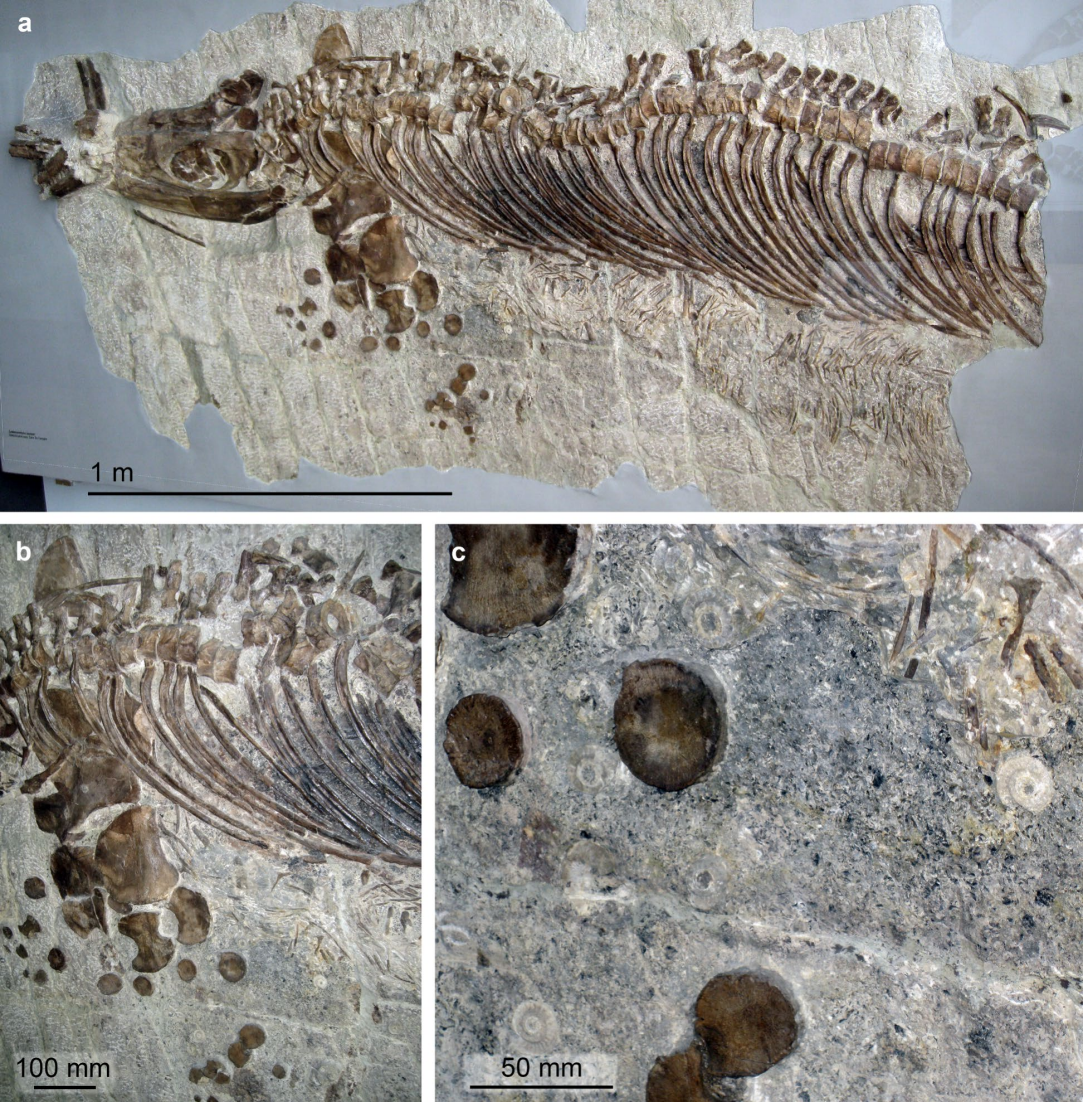
*Cymbospondylus buchseri* Sander, 1989, PIMUZ T 4351, Holotype, Besano Fm., Anisian, Middle Triassic, Cava Tre Fontane TI, on display in the Natural History Museum of the University of Zurich. **a** The entire skeleton. **b** Detail showing the fins and parts of the thorax with stomach content. **c** Detail of the stomach content with some phalanges and ammonoids (probably not part of the stomach content)

Material: Holotype PIMUZ T 4351 (Fig. 8).

Locality: Cava Tre Fontane near Serpiano and Meride (Ticino)

Stratigraphic position: Besano Formation, Anisian, Middle Triassic

**Fig. 9:**
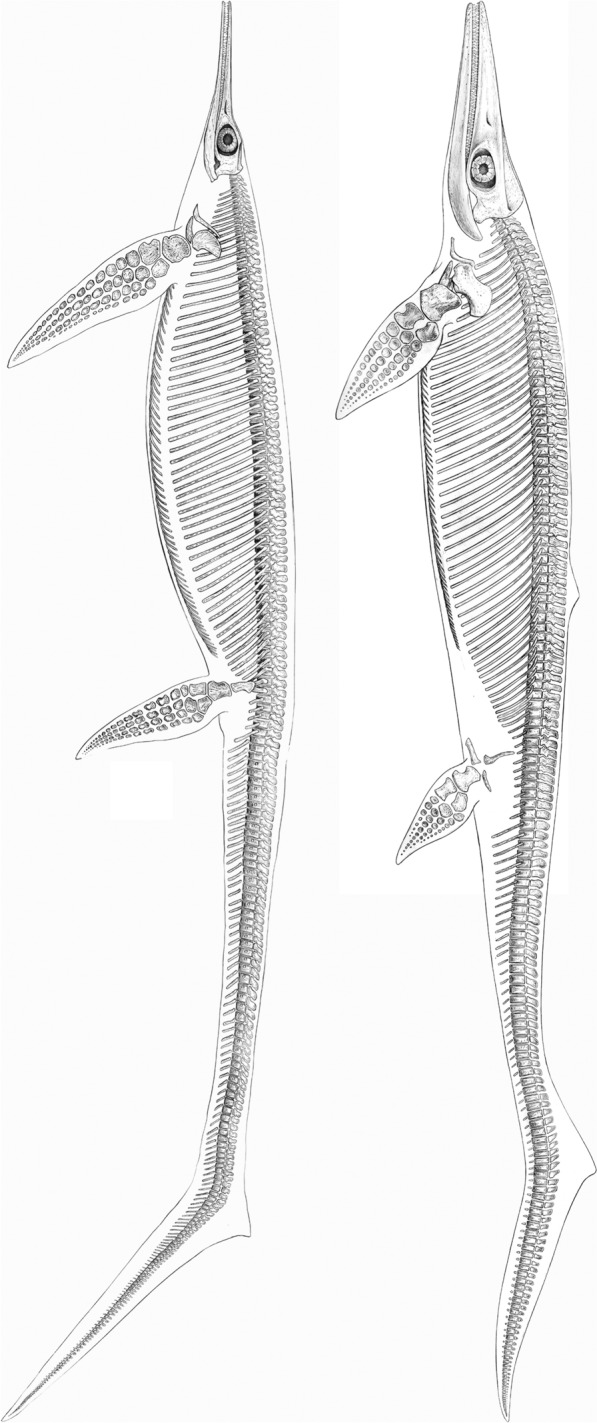
*Besanosaurus leptorhynchus* Dal Sasso & Pinna, 1996 (left) and *Cymbospondylus buchseri* Sander, 1989 (right), Anisian, Middle Triassic; skeletal reconstructions by Beat Scheffold; *Besanosaurus* after Nosotti and Teruzzi (2008: Fig. 12B) and Bindellini et al., (2024:Fig. 11)

Short description: The holotype and only referred specimen shows the anterior half of the skeleton with three-dimensionally preserved bones. The posterior half was lost during mining. The body length of *C. buchseri* can be reconstructed to about 5.5 m (Rieppel, 2019; Sander, 1989). However, bone histology suggests that the holotype animal was still growing at time of death (Sander, 1989). *C. buchseri* had a comparatively high and broad rostrum (see reconstruction in Fig. 9), and a long and slender body. The caudal fin was likely poorly developed as in *C. petrinus* (McGowan & Motani, 2003; Merriam, 1908).

Remarks: While the osteologically immature holotype reached only about 5.5 m, other members of the genus are estimated to have reached 17 m body length (Sander et al., 2021). McGowan and Motani (2003) mention that the skull is also poorly ossified, which is typical for immature individuals.

As in several other ichthyosaur fossils, especially from the Jurassic (Delsett et al., 2016; Wahl, 2009), the snout of the holotype is severely fractured. As demonstrated by Wetzel and Reisdorf (2006), ichthyosaur carcasses usually sank head-first. The pointed rostrum would have penetrated more or less deeply into the soft sediment on impact, and the force of impact is likely to have resulted in fracturing of the delicate rostral bones (Delsett et al., 2016; Wahl, 2009). We are unaware of other reasonable explanations of such snout deformations. To our knowledge, there is no documentation of any actualistic case of such cranial fracturing caused by an impact into the seabed. The stomach content (Fig. 9b, c) of the holotype comprises arm hooks and beaks of numerous phragmoteuthid coleoids (Brinkmann, 1997).

Merriamosauria Motani, 1999

Shastasauridae Merriam, 1895

*Besanosaurus leptorhynchus* Dal Sasso & Pinna, 1996

Figures 9, 10

**Fig. 10:**
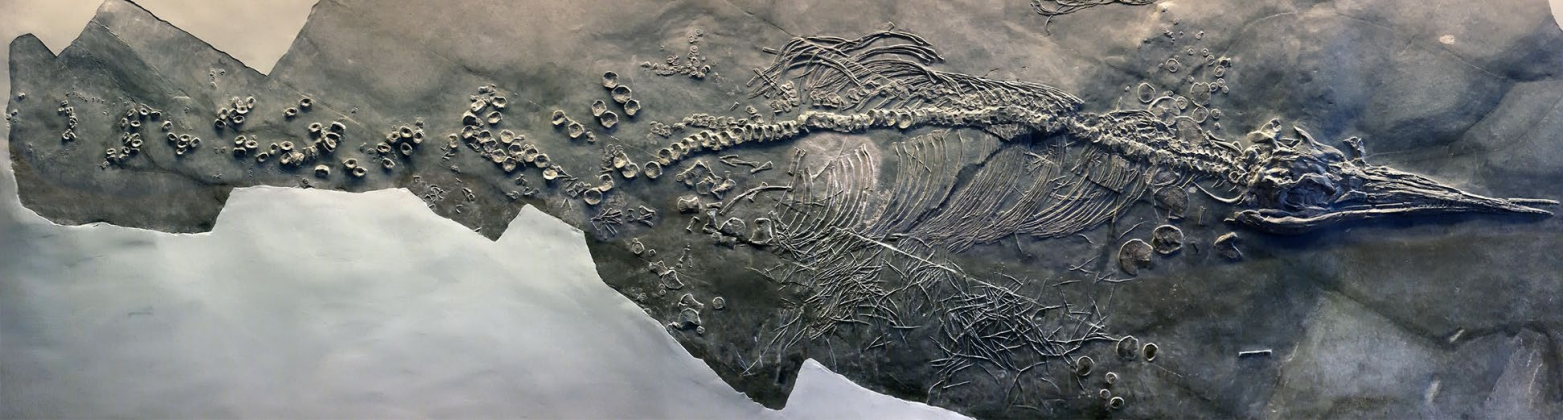
*Besanosaurus leptorhynchus* Dal Sasso & Pinna, 1996, PIMUZ T 4376, one of the most complete specimens of this species

Material: Holotype BES SC 999, PIMUZ T 4376 (Fig. 10) and additional materials (see Bindellini et al., 2021).

Locality: Holotype found near Besano (Italy), further specimens known also from localities close to Serpiano and Meride (Ticino)

Stratigraphic position: Besano Formation, Middle Triassic, Anisian

Short description: This ichthyosaur attained large sizes of up to 8 m body length (e.g., the strongly flattened specimen PIMUZ T4847). Several complete skeletons of juvenile individuals (Fig. 9) are available in Zurich and Milan. The species is characterized by an elongate, rather slender body and a slender snout with small teeth.

Remarks: *Mikadocephalus gracilirostris* (Maisch & Matzke, 1997) is a junior synonym of *Besanosaurus leptorhynchus* according to Bindellini et al. (2021).

Shastasauridae gen. et sp. indet.

Figures 11, 12

**Fig. 11:**
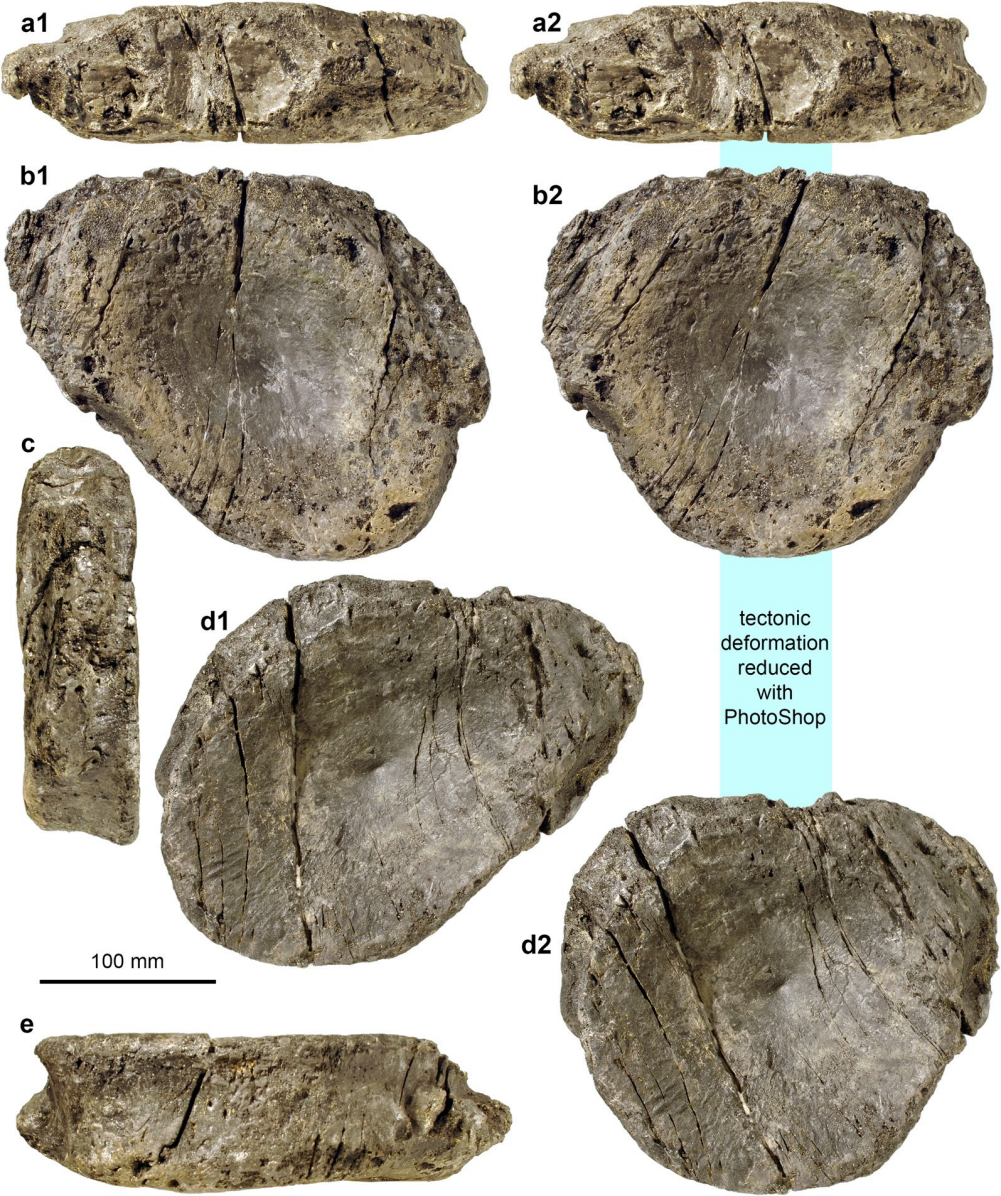
Shastasauridae sp. A. of Sander et al. (2022), dorsal vertebra, PIMUZ A/III 744a, Rhaetian, Schesaplana Member, Fil da Stidier ridge, Filisur, Grisons. **a** Dorsal; **b** posterior; **c** lateral, **d** ventral views. Figures labelled with 2 were retrodeformed using PhotoShop

**Fig. 12:**
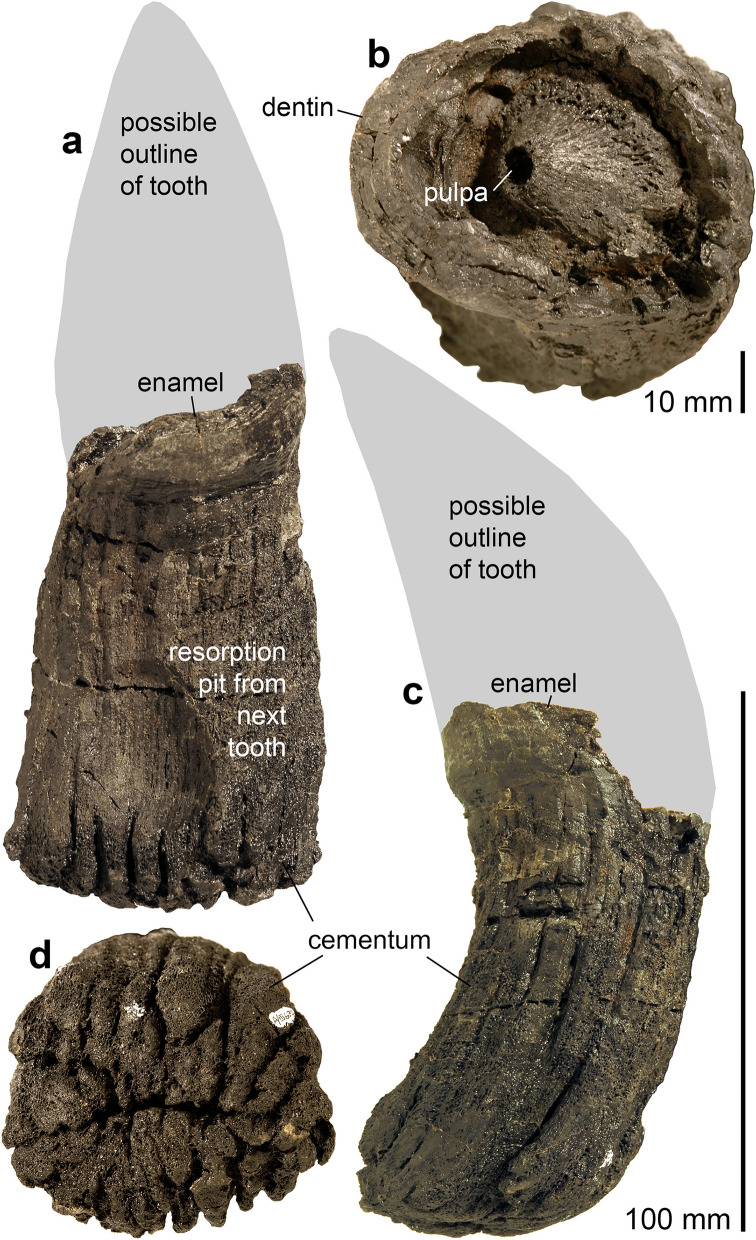
Fragmentary tooth (base and minor parts of the crown) of a giant shastasaurid PIMUZ A/III 670, Rhaetian Schesaplana Member, Crachenhorn Mountain, Davos-Monstein, Grisons. **a** Lingual; **b** apical; **c** mesial or distal; **d** basal views. In **a** and **c**, a possible tooth outline is reconstructed (grey surface)

Material: PIMUZ A/III 670, 744 (Figs. 11, 12) and additional referred materials (see Sander et al., 2022).

Locality: Graubünden

Stratigraphic position: Kössen Formation (Schesaplana Member), Late Norian to Rhaetian, Triassic

Short description: The Swiss specimens are isolated bones, teeth and disarticulated parts of skeletons including a large vertebra and several ribs (PIMUZ A/III 744). A fragmentary tooth (PIMUZ A/III 670), interpreted herein as also belonging to a shastasaurid (Figs. 11, 12), preserves a diameter of about 50 mm and had a reconstructed apicobasal height of 150 to 200 mm (see tentative reconstruction in Fig. 12a, c). The vertebra (PIMUZ A/III 744a; Fig. 11) associated with the ribs measures about 250 mm in diameter and suggests a body length of up to 20 m, similar to that of *Shonisaurus* (Nicholls & Manabe, 2004). Shastasauridae is famous for the giant adult body size of several of its genera and species, which belong to the largest ichthyosaurs known (Nicholls & Manabe, 2004; Sander et al., 2021).

Remarks: Triassic ichthyosaurs are known for both their diversity and their disparity in body size. Camp (1976, 1980), Nicholls and Manabe (2004), and Kelley et al. (2022) reported giant shastasaurids from North America, which reached approximately 20 m body length. Some decades ago, remains of huge latest Triassic (Rhaetian) shastasaurids were found in the mountains of eastern Switzerland; these remains have recently been described along with other Shastasauridae vertebrae from the upper Norian-lower Rhaetian Alplihorn Member of the Kössen Formation (Furrer, 1993; Sander et al., 2022). In contrast to *Shonisaurus sikanniensis* (Nicholls & Manabe, 2004) but similar to *S. popularis* (Kelley et al., 2022), at least some Swiss shastasaurids had huge teeth (Fig. 12).

### Jurassic

Parvipelvia Motani, 1999

Ichthyosauridae Bonaparte, 1841

*Protoichthyosaurus* Appleby, 1979

*Protoichthyosaurus* cf. *applebyi* Lomax et al., 2017

Figure 13

**Fig. 13:**
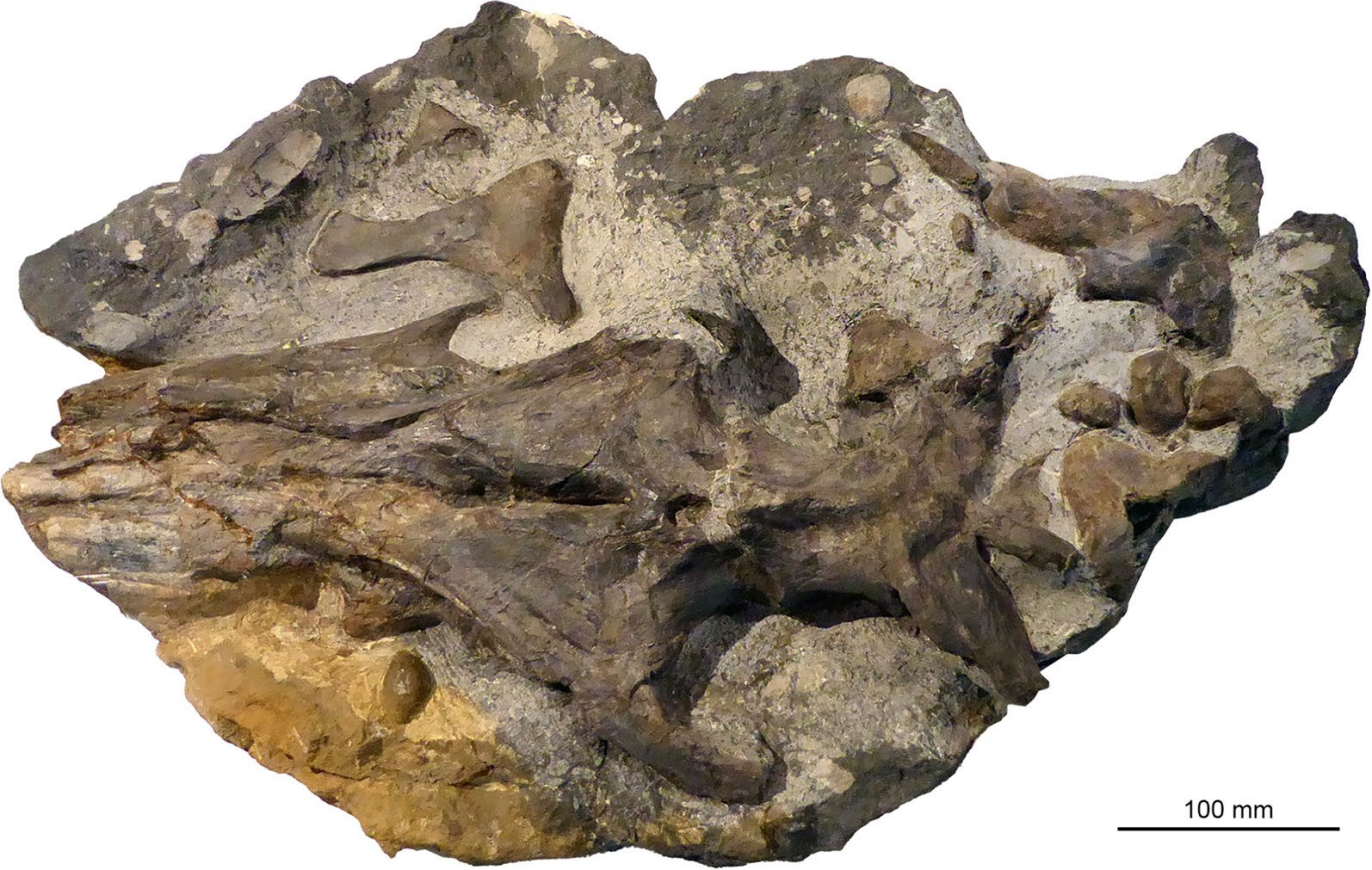
*Protoichthyosaurus* cf. *applebyi* Lomax et al., 2017, SMF 46, incomplete skull, dorsal view, upper Beggingen Member (“Arietenkalk”), semicostatum ammonite zone, Gruhalde, Frick AG

Material: SMF 46 (Fig. 13).

Locality: Frick AG, possibly also large specimens from Grellingen BL.

Stratigraphic position: Beggingen Member, Staffelegg Formation, Lower Sinemurian, Jurassic

Short description: The Frick skull is three-dimensionally preserved but lacks much of the snout (Maisch et al., 2008). *Protoichthyosaurus* is a moderate-sized ichthyosaur with robust dentition, a rostrum that is shorter than in more derived genera such as *Stenopterygius* or *Eurhinosaurus*, a dorsal region intermediate in length between, e.g., *Cymbospondylus* and *Ophthalmosaurus*, and moderately long forefins with three elements in the proximal carpal row. *Protoichthyosaurus* has two named species, *P. applebyi* and *P. prostaxalis* (Lomax et al., 2017). Only two specimens of *P. applebyi* are known, reaching a skull length of ~ 40 cm; however, the more abundant species *P. prostaxalis* could reach skull lengths of 80 cm (Lomax et al., 2019).

Remarks: The Frick specimen was originally referred to *Ichthyosaurus communis* by Maisch et al. (2008). Since then, our understanding of ichthyosaur diversity from the Hettangian-Sinemurian interval has changed drastically, with specimens previously referred to *Ichthyosaurus* having been split into two genera and eight species (Lomax & Massare, 2017; Lomax et al., 2017). Based on the participation of the parietal in the parietal foramen, as described by Maisch et al. (2008), the Frick specimen is inconsistent with *Ichthyosaurus* (see Lomax et al., 2020), but *Protoichthyosaurus* remains a possibility. Based on the limited exposure of the maxilla ventral to the nares, the Frick skull is most likely attributable to *P.* cf. *applebyi* (Lomax & Massare, 2018; Lomax et al., 2017, 2020). The specimen from Frick (Fig. 13) was probably much larger than documented specimens of *P. applebyi*, with a skull length estimated at 60 cm (Maisch et al., 2008), but within the range of *Protoichthyosaurus* skulls. Isolated bones that, based on their size may also belong to this taxon, occur occasionally in other Swiss localities (Peyer & Köchlin, 1934; Maisch et al. 2008).

Temnodontosauridae

*Temnodontosaurus* Lydekker, 1889

?*Temnodontosaurus* sp.

Figure 14

**Fig. 14:**
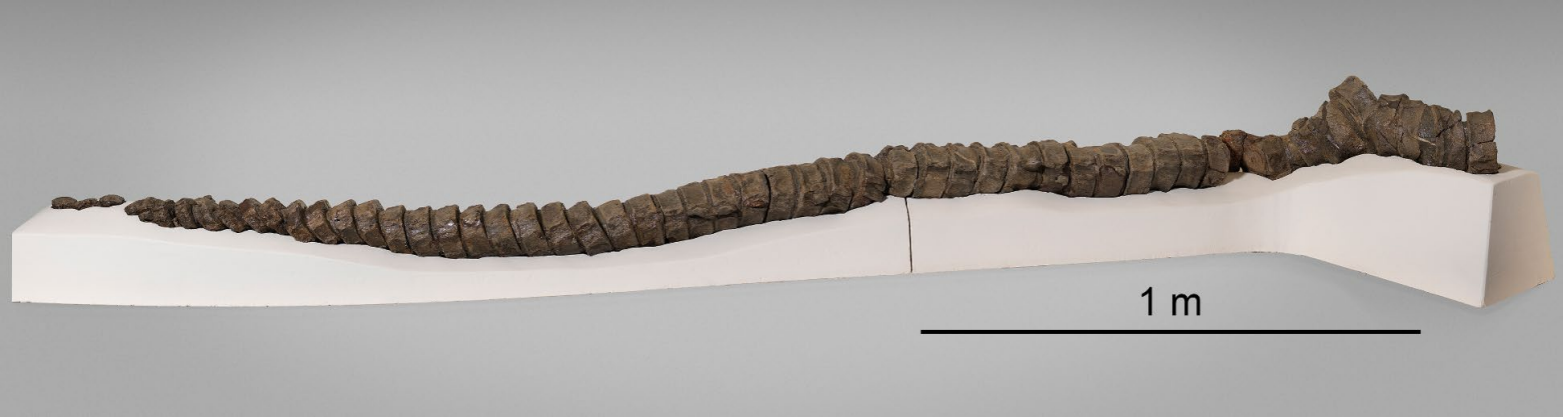
?*Temnodontosaurus* sp., NAT19310.001-.003, Staffelegg Formation, Lower Sinemurian, Beggingen (Schaffhausen). The vertebral column is on display in the Museum Allerheiligen SH. Photo by Urs Weibel

Material: NAT19310.001-0.003 (Fig. 14).

Locality: Beggingen (Schaffhausen).

Stratigraphic position: Beggingen Member, Staffelegg Formation, Lower Sinemurian, Lower Jurassic

Short description: A partial caudal skeleton (NAT19310.001-0.003) was referred to ?*Temnodontosaurus* based on the dimensions of the vertebrae. *Temnodontosaurus* is a large ichthyosaur (up to 15 m in length: McGowan, 1996) with robust jaws and skull. The forefin has three digits and one postaxial accessory digit.

Remarks: NAT19310.001-0.003 was excavated by Früh (1962) in the 1960s. It is tentatively included in ?*Temnodontosaurus* because of the large size of the vertebrae (up to 120 mm in diameter) and stratigraphic age. While the Sinemurian-aged *Leptonectes solei* has equally large anterior caudal vertebrae (~ 140 mm in diameter in the holotype), the vertebrae of this species are proportionately longer than those of the Schaffhausen specimen (EEM, pers. observ.), making a referral to *Temnodontosaurus* more plausible. The vertebrae are on display in the Museum Allerheiligen in Schaffhausen.

Leptonectidae Maisch 1998

*Eurhinosaurus* Abel, 1909

*Eurhinosaurus longirostris* (Mantell, 1851)

Figure 15

**Fig. 15:**
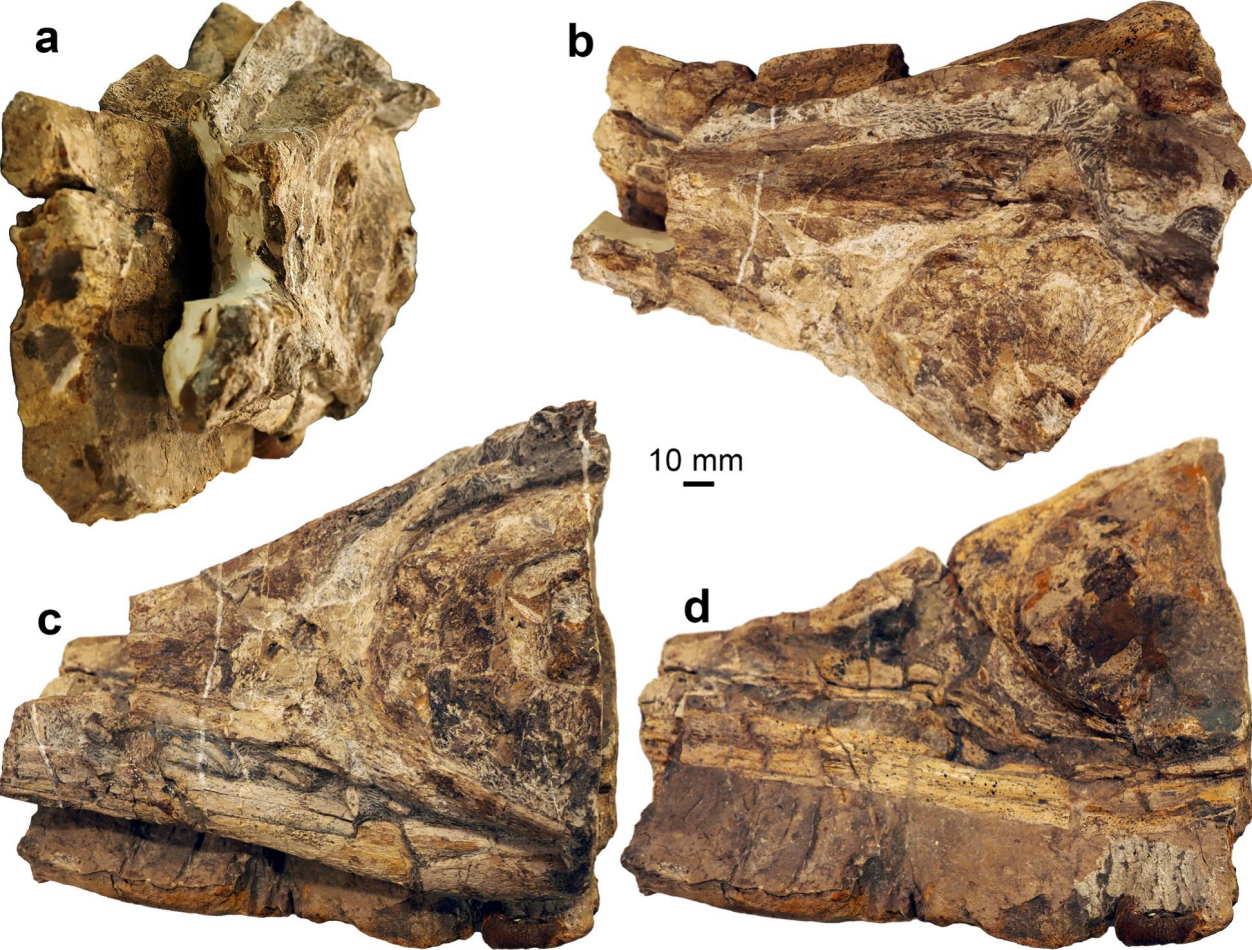
*Eurhinosaurus longirostris* (Mantell, 1851), PIMUZ A/III 749, Rietheim Member, Toarcian, Early Jurassic, Staffelegg (Aargau), photos were taken by Alexandra Wegmann (Zurich)

Material: PIMUZ A/III 749 (Fig. 15), on display at the Naturama in Aarau.

Locality: Staffelegg AG

Stratigraphic position: Rietheim Member, Toarcian, Jurassic

Short description: The specimen from Staffelegg is a slightly deformed incomplete skull, which preserves the huge orbits with the sclerotic ring and the base of the very slender rostrum (Reisdorf et al., 2011). *Eurhinosaurus* is one of the most remarkable ichthyosaurs because of its outstanding morphology with an extremely elongate, tooth-bearing upper jaw that is about twice as long as the lower jaw (e.g., Maisch & Matzke, 2000; McGowan & Motani, 2003); its huge orbits give the skull a mosquito-like appearance. Also, the slender body of this genus reached impressive lengths of just over 7 m (McGowan & Motani, 2003), with long paired fins (e.g., Maisch & Matzke, 2000).

Remarks: Jobbins et al. (2024) compared this ichthyosaur with other vertebrates with extremely elongated upper or lower jaws because of its pronounced overbite (e.g., Maisch & Matzke, 2000; McGowan & Motani, 2003). This discrepancy in upper versus lower jaw length may be linked to “to strike and confuse prey” (Jobbins et al., 2024: p. 11).

Parvipelvia, Unnamed clade (Maxwell & Cortés, 2020)

*Hauffiopteryx* Maisch, 2008

*Hauffiopteryx typicus* (von Huene, 1931)

Figure 16

**Fig. 16:**
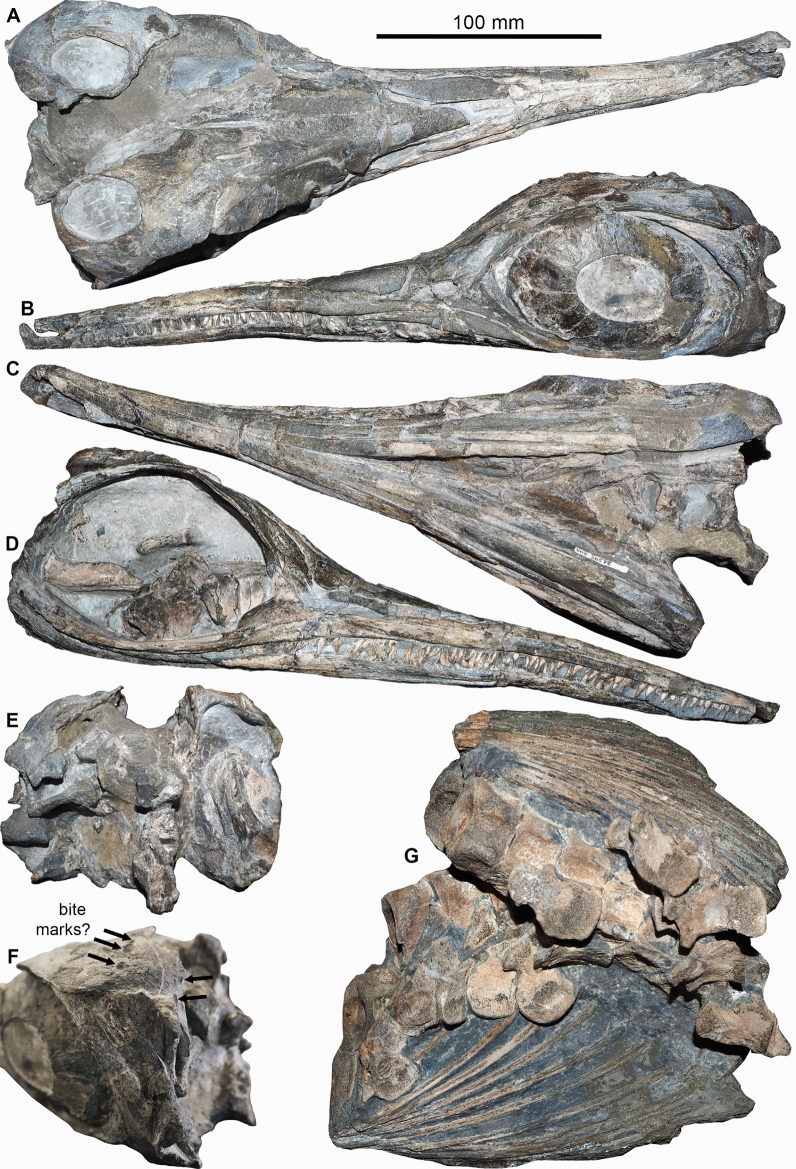
*Hauffiopteryx typicus* (von Huene, 1931), NMO 26575, Pliensbachian, Unterer Hauenstein (Solothurn). **A** dorsal; **B** left lateral; **C** ventral; **D** right lateral; **E** posterior view of the articulated skull. **F** Detail showing potential bite marks on the supratemporal. **G** Postcranial elements including about ten thoracal vertebrae, neural arches, thoracal ribs and a few pectoral fin elements

Material: NMO 26575 (Fig. 16).

Locality: Unterer Hauenstein SO

Stratigraphic position: Müsenegg Bed, Breitenmatt Member, Staffelegg Formation, Pliensbachian, Jurassic

Short description: The specimen NMO 26575 was embedded in a quite common position with the skull vertically sticking in the sediment (Maisch & Reisdorf, 2006a, b). The anterior postcranium was oriented subvertically behind it. It is limited to some articulated vertebrae, neural arches, ribs, gastralia, and phalanges. The rest of the postcranium likely came to rest on the sediment surface. *Hauffiopteryx* reached a body length of up to 3 m (Maxwell & Cortés, 2020). Its skull bore a short and quite slender rostrum (upper jaw slightly longer than the lower jaw) and big eyes. The dorsal region is moderately short and moderately slender (between *Cymbospondylus* and *Ophthalmosaurus*). The tail is approximately as long as the dorsal region and bears a narrow symmetrical caudal fin.

Remarks: The specimen was originally referred to the Hettangian-Sinemurian species *Leptonectes tenuirostris* by Maisch and Reisdorf (2006a, b), although inconsistencies in phalangeal shape were noted. This aspect, in addition to details of skull morphology, led to the specimen being reassigned to the Toarcian species *Hauffiopteryx typicus* by Maxwell and Cortés (2020). Remarkably, the vertically embedded skull and the surrounding concretion at least partially diagenetically penetrated three ammonite zones (Wetzel & Reisdorf, 2006). Both the posterior skull and the postcranium were exposed to scavenging over a prolonged time. The left supratemporal bears five holes (Fig. 16F), which lack indications for healing. It is unclear, what made these holes, but it was quite likely post mortem, which fits with the vertically embedded skull and the posterior being exposed over a long time. Superficially, the holes resemble bite traces produced by reptile predators or scavengers (e.g., Scheyer et al., 2024), but they may as well have formed by erosion or some diagenetic process (future examination might clarify the origin of these holes). It is also noteworthy that several specimens of the minute gastropod *Coelodiscus* accumulated between the postcranial elements.

Baracromia Fischer et al., 2013.

Stenopterygiidae Woodward in von Zittel, 1932.

*Stenopterygius* Jaekel, 1904, emend. Von Huene, 1922.

*Stenopterygius* sp.

Figures 17, 18

Material: Nearly complete skeleton NMBE5014842. Bone-bearing slabs MGL 42002, 42003, 42004, 40947, 40950, 40948, 40949 (Weidmann, 1981).

Locality: Creux de l’Ours, Teysachaux FG; Le Ruisseau du Chalevay, South of Col de Soladier, 3 km northeast of Avants/Montreux, VD; Asuel JU.

Stratigraphic position: Teysachaux FG—Soladier Member, Staldengraben-Formation, and Asuel JU—Rietheim Member, Staffelegg Formation, Toarcian, Early Jurassic.

**Fig. 17:**
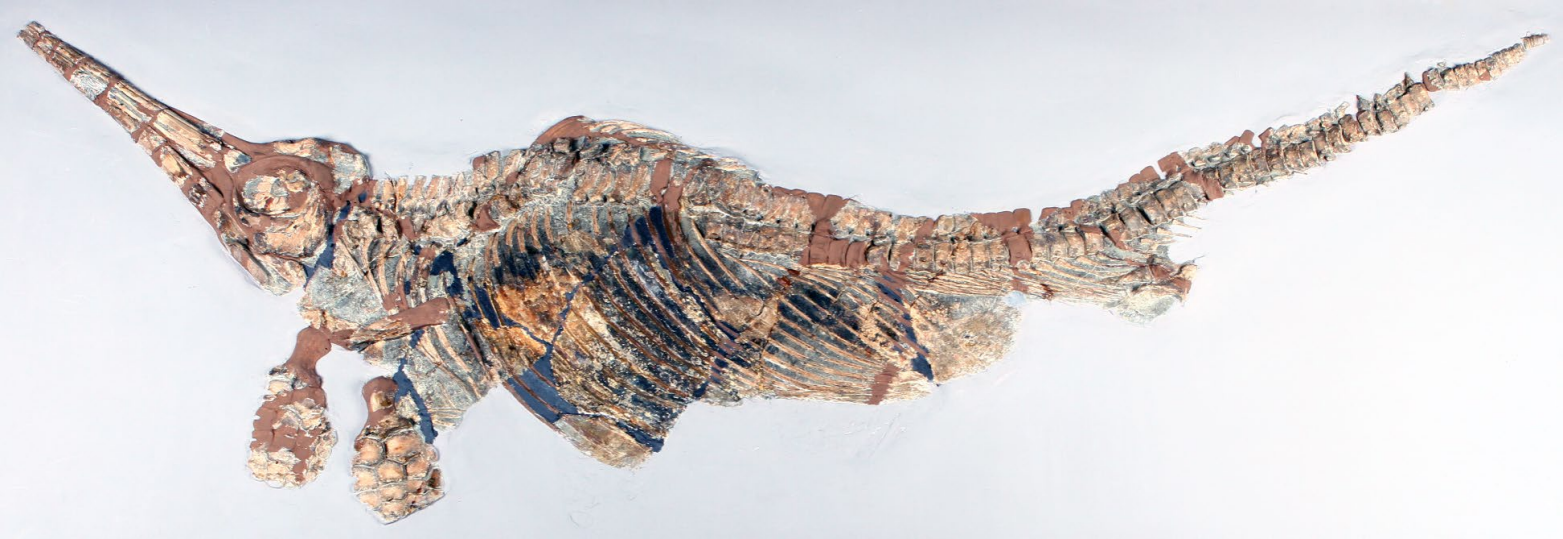
*Stenopterygius* sp., NMBE5014842 (inventarized in Bern, on display in Fribourg), Soladier Member, Staldengraben-Formation, Toarcian, Teysachaux FG, photo by Hans-Rüdiger Siegel

**Fig. 18:**
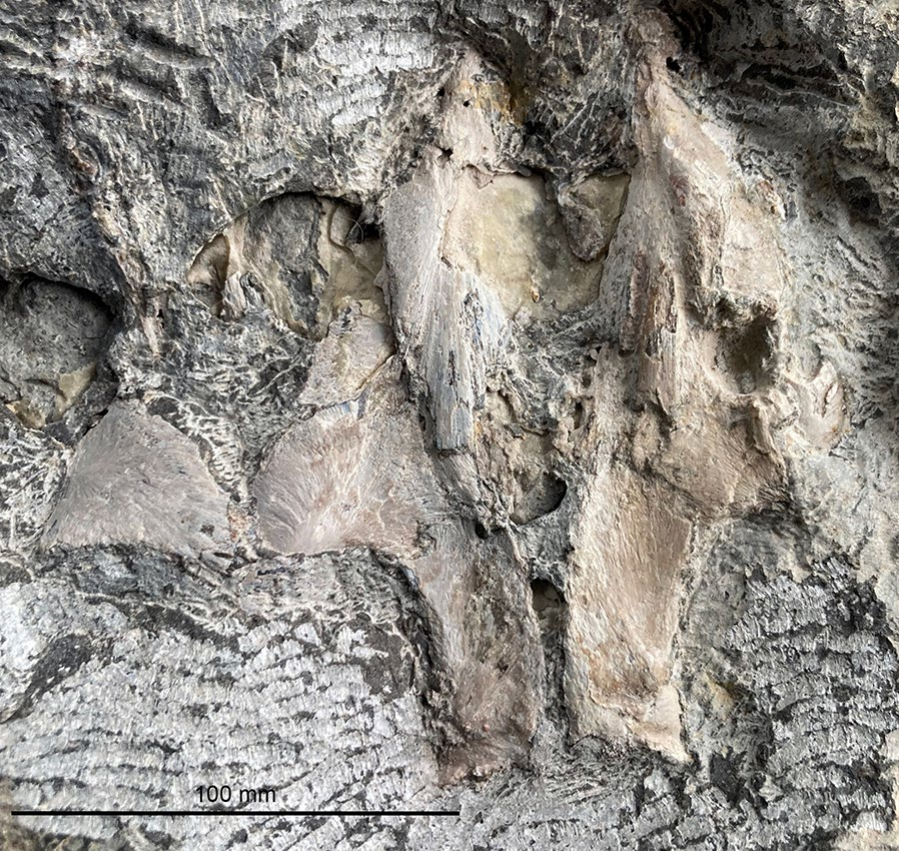
?*Stenopterygius* sp., Rietheim Member, Staffelegg-Formation, Toarcian, Asuel JU, photo by Bernhard Hostettler

Short description: The skeleton NMBE5014842 from Teysachaux FG (Fig. 17) is nearly complete and over 2 m long, although poorly preserved (von Huene, 1939). A much more fragmentary specimen (MGL 42002, 42003, 42004, 40947, 40950, 40948, 40949) may also belong to *Stenopterygius* and was briefly described by Weidmann (1981). It comprises three vertebrae between 36 and 45 mm in diameter and remains of at least 15 ribs. It lacks clearly diagnostic bones. A nearly complete skull with three-dimensionally preserved bones in a nodule was found in a valley near Asuel (Fig. 18). The nodule with the skull is only partially prepared, and hence the assignment is somewhat uncertain. *Stenopterygius* is a mid-sized ichthyosaur with adults between 2.0 and 3.75 m in length, characterized by a long rostrum (almost as long as in *Ophthalmosaurus*) with variably reduced teeth, and a moderately regionalized vertebral column.

Remarks: NMBE5014842 was acquired from the finder Joseph Cardinaux in 1870 by the Natural History Museum of the Burgergemeinde Bern for 120 Francs. In 1934, the numerous fragments of the huge C-shaped concretion were sent to Bernhard Hauff in Holzmaden, Germany, who was then considered the most experienced preparator. Von Huene (1939), also based in Germany, described this specimen, which is mainly remarkable for its geographic origin. This might be the source of the rumour that the skeleton might be German rather than Swiss. Furrer (1960) and Menkveld-Gfeller (1998) provided numerous lines of independent evidence that the skeleton indeed comes from the alpine equivalent of the Posidonienschiefer Formation (Soladier Mb.) in the Teysachaux region. Due to extensive reconstruction and poor preservation, the specimen cannot be referred to species level (Maisch, 2008).

Ophthalmosauria Motani, 1999

*Argovisaurus* Miedema et al., 2024

*Argovisaurus martafernandezi* Miedema et al., 2024

Figures 19, 20)

Material: Holotype PIMUZ A/III 5279.

Locality: Auenstein, Oberegg quarry AG

Stratigraphic position: Lower Acuminata beds, Hauptrogenstein Formation, Middle Jurassic, *subfurcatum/ niortense* zone of the Middle Bajocian (Meyer, 1988).

Short description: The disarticulated skull and most of the dorsal region are known based on the holotype and only known specimen; the limbs and tail are not preserved (Fig. 20). With a skull length of about 1.3 m, *Argovisaurus* was a large early-diverging ophthalmosaurian. Its skull bore robust jaws and moderately large eyes. It appears to have been rather deep-bodied.

Remarks: Based on taphonomic evidence such as disarticulation, traces of scavenging and oyster overgrowth on a vertebra, Miedema et al. (2024) indicate that the carcass was covered by sediment after a rather long exposure time. The excellent three-dimensional preservation of the bones permitted the reconstruction of the skull. This species is important because it was found in the stratigraphic interval (stage) with the poorest ichthyosaur record of the Jurassic (Fischer et al., 2021). Additionally, the *Argovisaurus* holotype is the largest and most complete skeleton of the Bajocian. It is of special importance because of its position at the base of the Ophthalmosauria (Miedema et al., 2024), the clade to which most Late Jurassic and Cretaceous ichthyosaurs belong, that originated in the early Middle Jurassic.

**Fig. 19:**
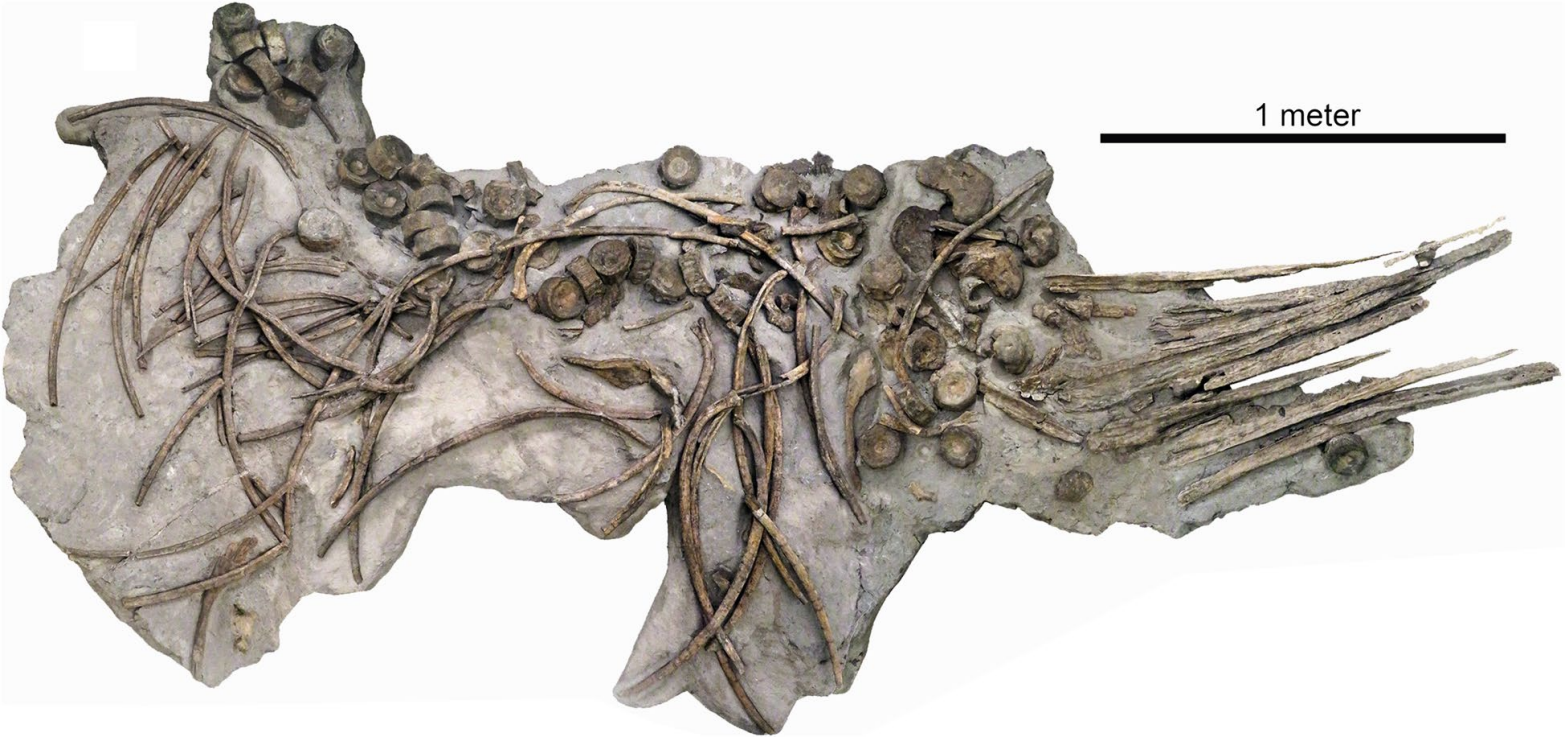
*Argovisaurus martafernandezi* Miedema et al., 2024, PIMUZ A/III 5279, Bajocian, Auenstein, Oberegg quarry (Aargau)

**Fig. 20:**
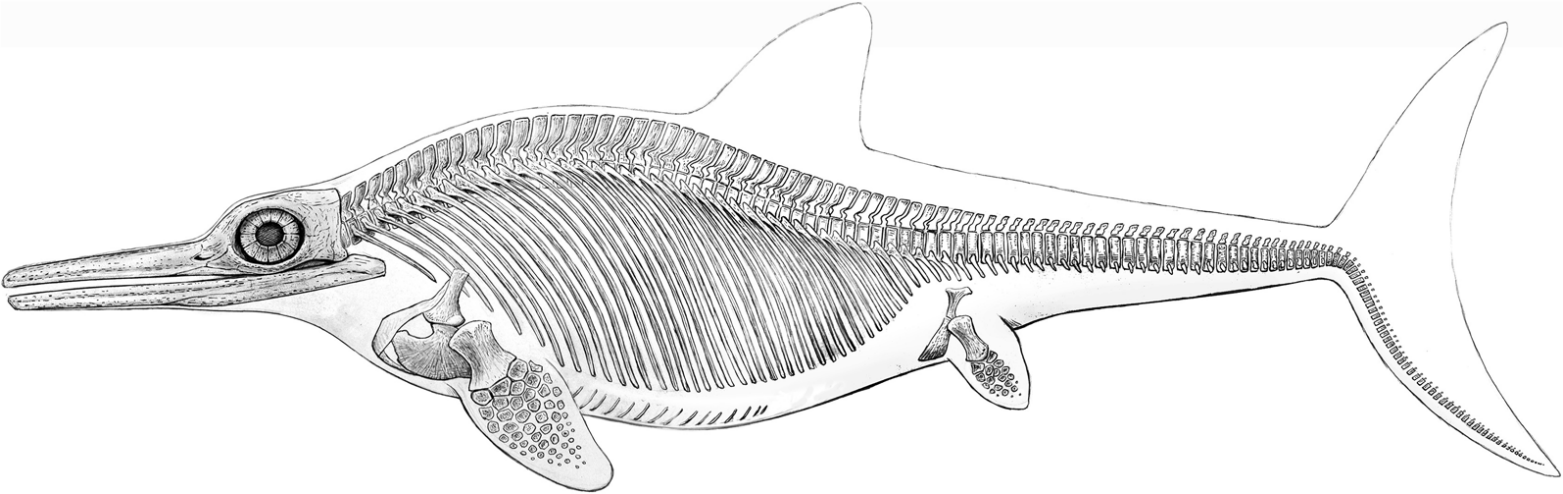
*Argovisaurus martafernandezi* Miedema et al., 2024, skeletal reconstruction by Beat Scheffold

Platypterygiinae

Figure 21

**Fig. 21:**
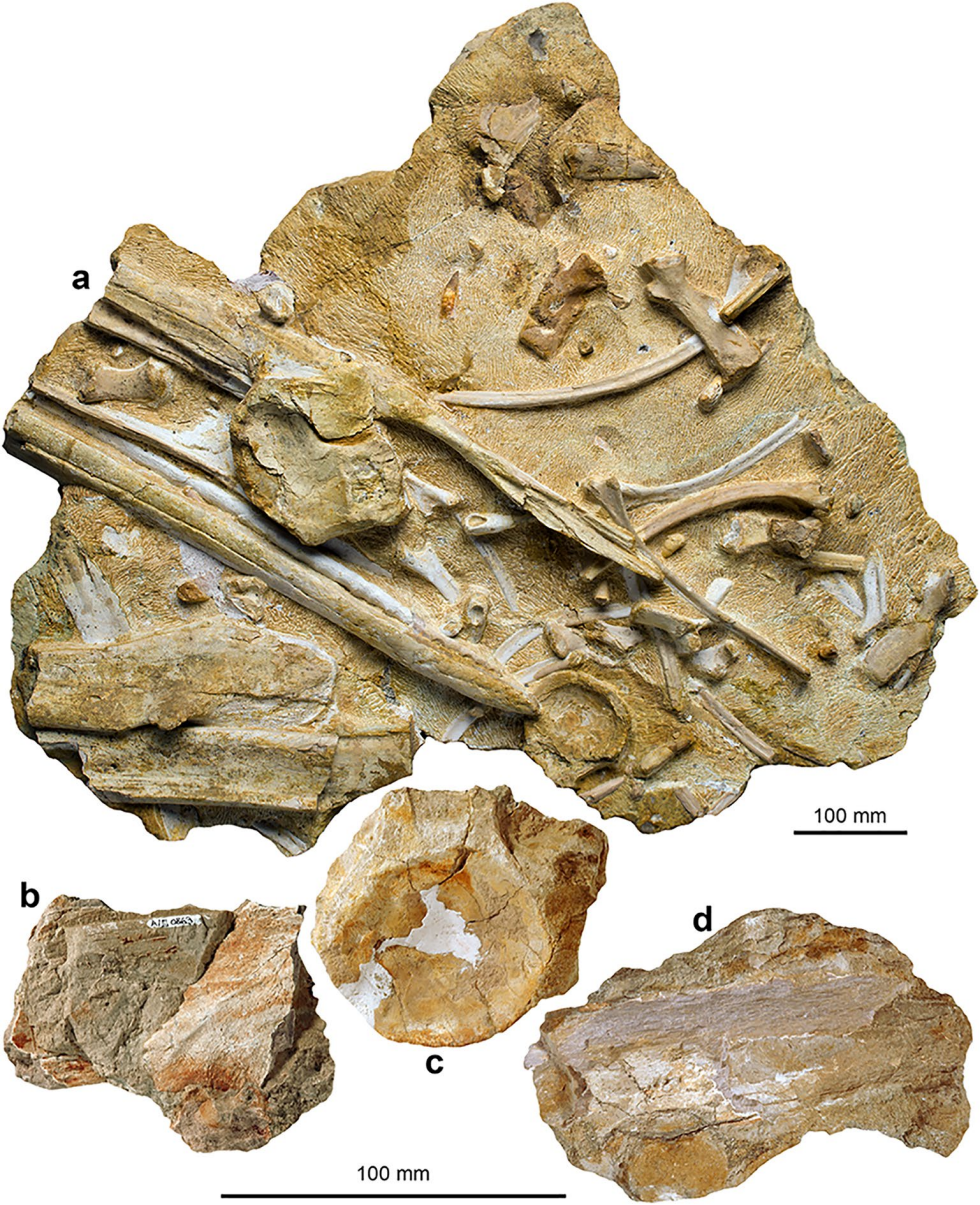
Skeletal remains of Platypterygiinae indet. from the Kimmeridgian of the Born near Ruppoldingen SO. **a** NMO-26734, disarticulated partial skeleton. **b** to **d** PIMUZ A/III 863, isolated bones (skull remains and a vertebra)

Material: NMO-26330, -26,734 (Fig. 21). Dubbed «Bornsaurier» because of its origin.

Locality: Born near Ruppoldingen SO

Stratigraphic position: Wettingen Member, Burghorn Formation, Kimmeridgian, Jurassic

**Fig. 22:**
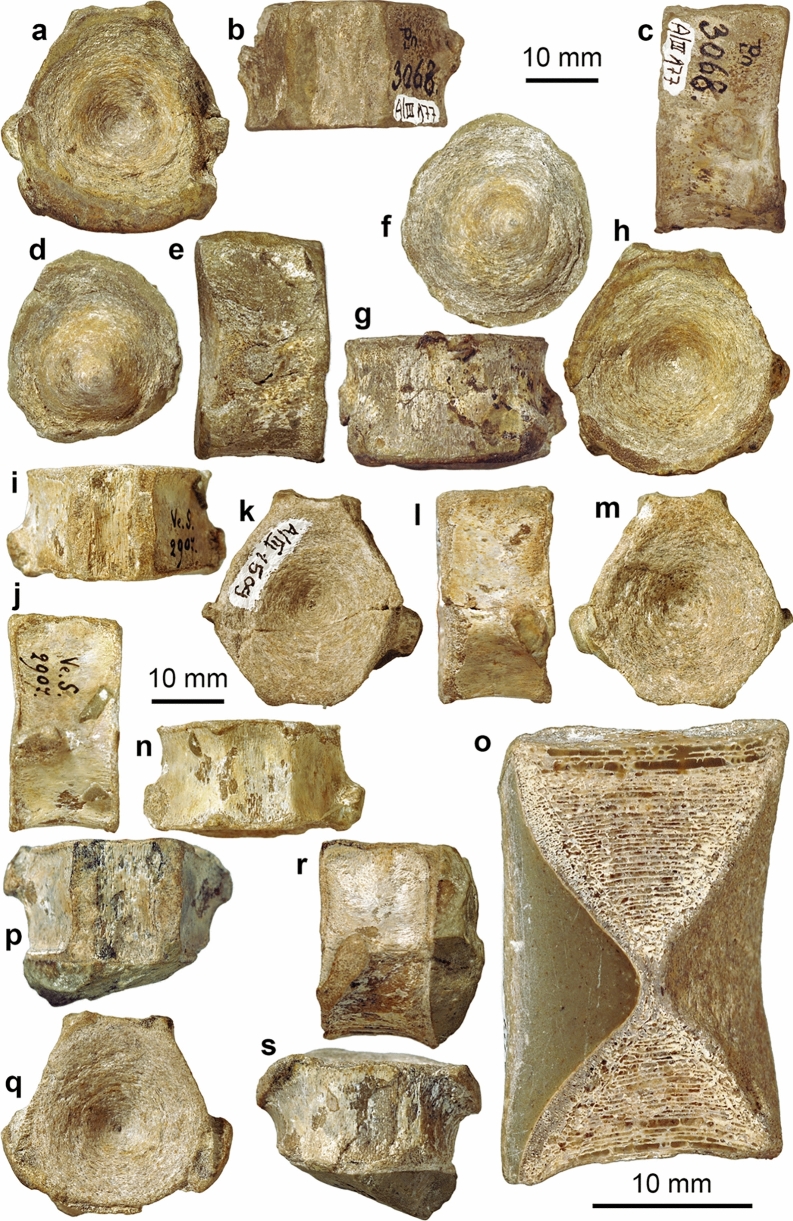
Isolated vertebra centra of **“***Mixosaurus helveticus”* (**a**–**o**) and *Cymbospondylus* sp. (**p**–**s**), Wellendolomit Member, Lower Muschelkalk Group, Laufenburg AG. **a**–**h** PIMUZ A/III 177, showing all sides and the external moulds of both facets. **i**–**m** PIMUZ A/III 1509. **o** PIMUZ A/III 1508, vertically sectioned vertebra. **p**–**s** PIMUZ A/III 175

Short description: The main slab (Fig. 22a) preserves some of the extremely slender jaw bones, teeth, a left quadrate in lateral view, as well as vertebrae and ribs. The tooth roots are quadrangular in cross-section, allowing referral to Platypterygiinae (Fischer et al. 2021). Platypterygiine ichthyosaurs are characterized by broad forefins and moderate body sizes of up to 7 m in length; within the clade there is relatively high anatomical disparity.

Remarks: Generally, the post-Toarcian fossil record of ichthyosaurs is meagre in Switzerland. The partial skeleton from the Born near Ruppoldingen (NMO-26734) is currently the only identifiable ichthyosaur from the Late Jurassic of Switzerland. Remarkably, it was kept unprepared since 1905 in the Naturmuseum Olten, where it was over many years considered to be crocodile remains. Maisch (2014) described it as *Brachypterygius mordax*, a presumed error since he went on to state that *B. extremus* and *Grendelius mordax* represented the same taxon, and therefore the Swiss specimen should have been referred to the senior synonym *B. extremus*. Because of the quadrangular tooth roots, the specimen can confidently be referred to the ophthalmosaurian subfamily Platypterygiinae, which includes *Brachypterygius/Grendelius*. However, several Late Jurassic taxa with quadrangular tooth roots have been documented, including *B. extremus/G. mordax*, *G. alekseevi, Acuetzpalin carranzi,* and *Undorosaurus* spp. (see Barrientos-Lara & Alvarado-Ortega, 2021). Thus, this character cannot be considered diagnostic at even the generic level.

The collection of the University of Zurich also keeps some isolated bone fragments (Fig. 21b–d), which perhaps belong to the same taxon. The locality further yielded remains of chondrichthyans, marine crocodiles and pliosaurids.

### Cretaceous

Isolated remains have been recovered from the Cretaceous of Switzerland (see below), but to date no remains diagnostic to genus level have been described. See the section below.

### Fragments unidentifiable on genus and species level

Because of the fragmentary nature of the materials listed below, we refrain from providing descriptions of the entire animal.

Mixosauridae indet.

Figure 22

**Fig. 23:**
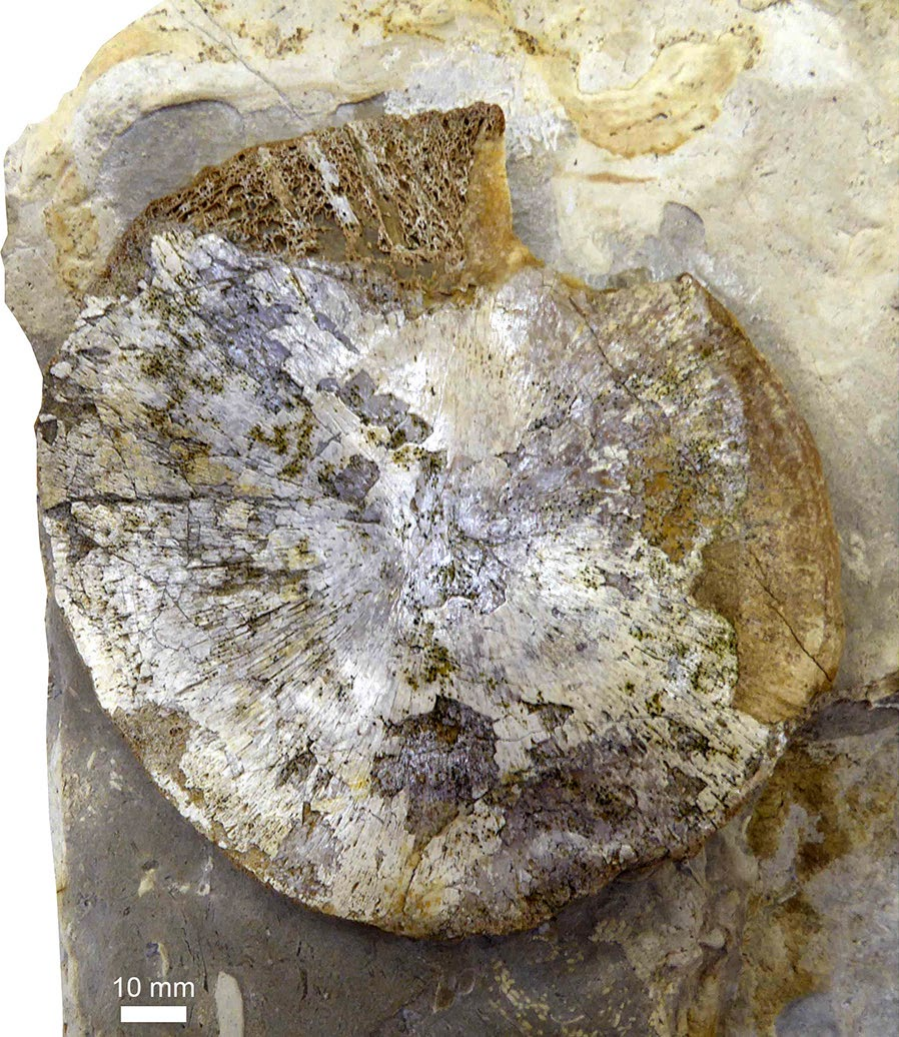
Humerus or coracoid of a ?shastasaurid from the Trochitenkalk (lower part of the Upper Muschelkalk Group), Nat19309, NE of Schleitheim. Left: after preparation. Right: unprepared

Material: PIMUZ A/III 175, 177, 1508, 1509 (Fig. 23).

Locality: Laufenburg AG

Stratigraphic position: Lower Muschelkalk, Anisian, Middle Triassic

Short description: The vertebrae in the collections of the University of Zurich display the characteristic slightly hexagonal outline of mixosaurid vertebrae and are of a moderately small size (up to 32 mm high).

Remarks: Von Huene (1916) described several isolated ichthyosaur vertebrae (on his plate V, some re-figured here in Fig. 22) stored in the collections of the Palaeontological Institute of the University of Zurich, for some of which he introduced the species “*Mixosaurus helveticus*”. McGowan and Motani (2003) follow Mazin (1983) in considering *M. helveticus* a *nomen dubium*. Centrum size and shape are reminiscent of a small mixosaurid like *M. cornalianus*, although this material is older than the material from the Besano Formation. A generic assignment is not possible given the lack of vertebral characters differentiating *Mixosaurus* and *Phalarodon*.

? Shastasauridae gen. et sp. indet.

Figures 23, 24

**Fig. 24:**
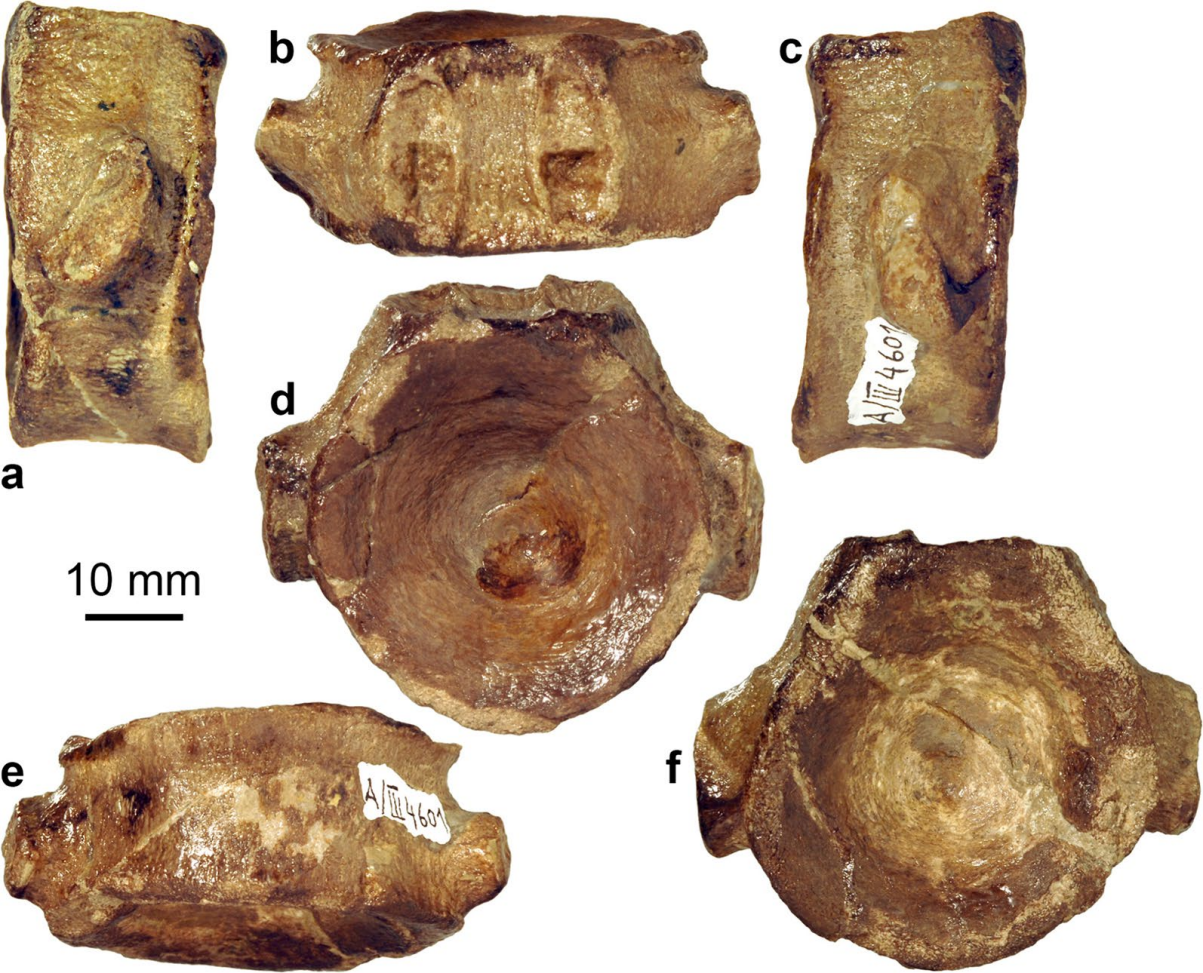
Vertebra of **a** ?shastasaurid (“*Pessosaurus suevicus*”), PIMUZ A/III 4601, Trochitenkalk (lower part of the Upper Muschelkalk Group), Auhald, Schleitheim. **a**, **c** Lateral; **b** dorsal; **e** ventral; **d**, **f** anterior and posterior views

Material: Nat19309 (Fig. 23), PIMUZ A/III 4601 (Fig. 24).

Locality: Mining area at the slope of the Wutach valley, ca. 1 km E of Schleitheim SH.

Stratigraphic position: Upper Muschelkalk Group, Schinznach Formation, “Trochitenkalk”, Anisian.

Short description: Nat19309 is a single subcircular bone measuring about 120 × 125 mm, which was collected from scree. It was acid prepared and shows the characteristic lateral concavity of a humerus, but it could also be a coracoid. One side is slightly corroded, showing the characteristically lightly built ichthyosaur spongiosa (Fig. 23).

Remarks: The specimen is kept at the Museum zu Allerheiligen in Schaffhausen. A vertebra had been assigned to “*Pessosaurus suevicus*” and is kept in Zurich with the number PIMUZ A/III 4601 (Fig. 24). “*Pessosaurus suevicus*” is a *nomen dubium*, with type material consistent with ?Shastasauridae indet. (Maisch & Matzke, 2000); the referred vertebra is consistent with this assessment.

*Ophthalmosauria* gen. et sp. indet.

Figure 25

**Fig. 25:**
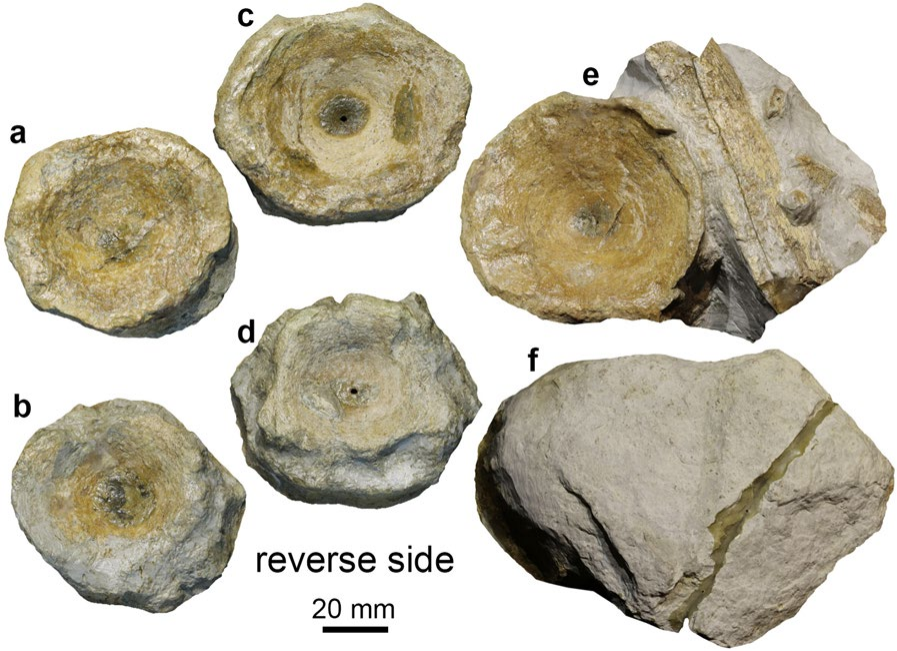
Ophthalmosauria gen. et sp. indet., NAT19310.001- to -003, Museum zu Allerheiligen, Schaffhausen, Schwarzbach Member, Kimmeridgian, quarry between Thayngen SH and Bibern SH

Material: NAT19310.001- to -003 (Fig. 25).

Locality: Abandoned quarry in Thayngen SH.

Stratigraphic position: Schwarzbach Member, Lower Kimmeridgian, Jurassic.

Remarks: Disarticulated skull remains of a moderately sized ophthalmosaurian (not figured) were recently discovered, which are neither prepared nor described (work in progress). A few vertebrae, possibly not from the same individual, were found several decades ago in the same stratigraphic interval, but at a distance of about 200 m (Fig. 25). One of the vertebrae is associated with a rib fragment. The specimen is at the Museum Allerheiligen in Schaffhausen.

Ophthalmosauria gen. et sp. indet.

(not figured)

Material: Worn tooth (PK 7B.05.04; Scheyer, 2018: fig. 730), worn vertebrae (NMSG P13598, PK 7B.32.05; Scheyer, 2018: figs. 731, 732).

Locality: Tierwis SG (PK 7B.05.04), Stütze 2 (Pillar 2) AR (NMSG P13598), Wildhuser Schafboden SG (PK 7B.32.05), Alpstein Massif

Stratigraphic position: Garschella Formation, Aptian, Cretaceous.

Remarks: Aptian sediments occasionally yielded isolated ichthyosaur elements such as a tooth and two corroded vertebrae, which are figured in Scheyer (2018). These are non-diagnostic and are referred to Ophthalmosauria solely based on stratigraphic criteria and the absence of other ichthyosaur clades in the Aptian.

Ophthalmosauria indet.

Figure 26

**Fig. 26:**
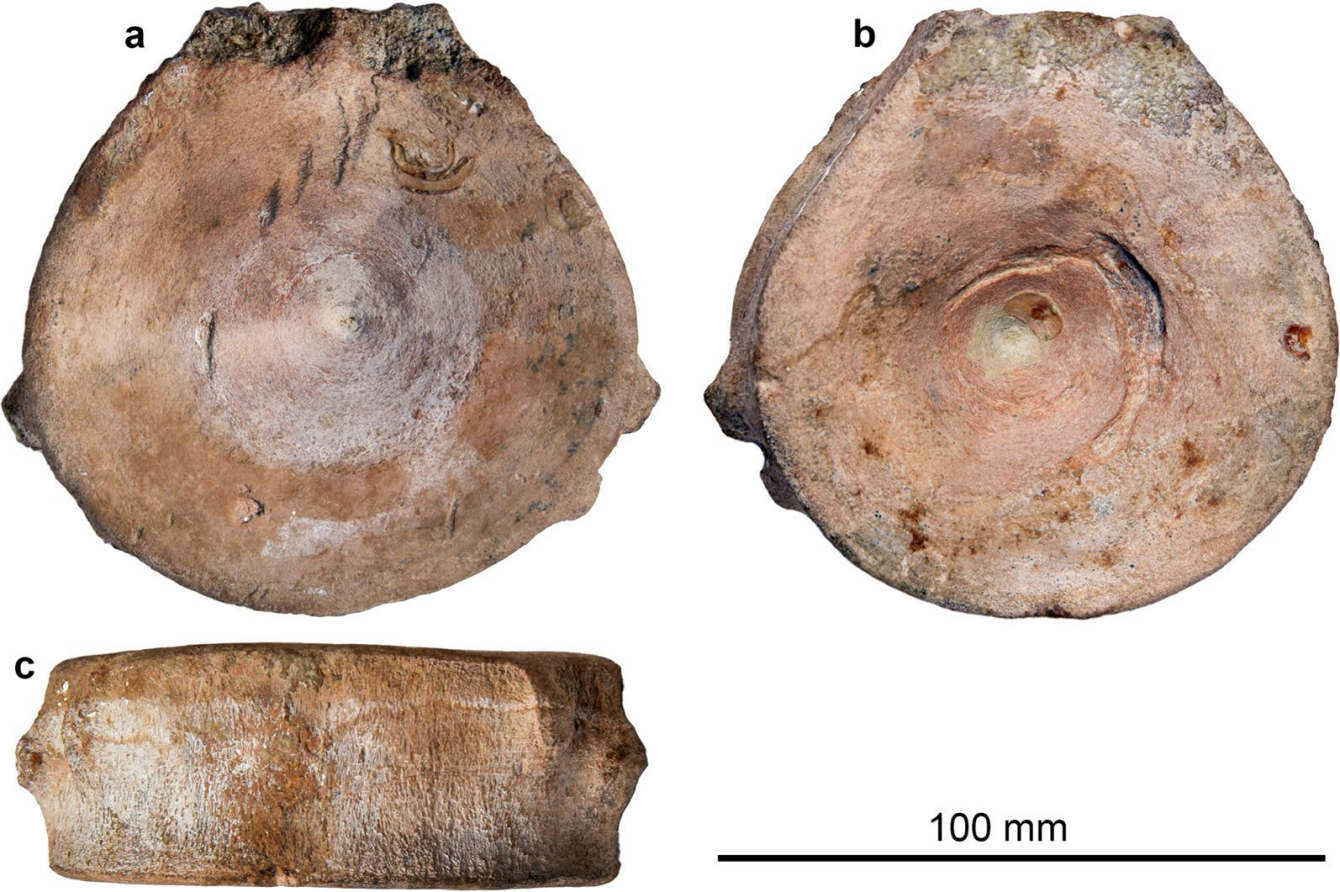
Ophthalmosauria indet., REG-28816, Aptian, La Presta (Neuchâtel). Photos provided by Thierry Malvesy (Neuchâtel)

Material: REG-28816 (Fig. 26).

Locality: La Presta NE

Stratigraphic position: Early Cretaceous, Aptian

Short description: We figure a well-preserved centrum from La Presta, which measures about 100 mm in diameter and belonged to an animal that likely was over 4 m long.

Remarks: This centrum represents the first published discovery of a Cretaceous ichthyosaur from the Swiss Jura (Ayer, 2003), although further isolated remains from the Cretaceous of other parts of Switzerland lie in museum collections (see above). The centrum was initially referred to *Platypterygius*, which at the time of publication, was the only accepted genus of Cretaceous ichthyosaur (Ayer, 2003). It is here referred to Ophthalmosauria based on its size and Cretaceous stratigraphic origin. The specimen belongs to the Muséum de Neuchâtel.

## Conclusions

Here, we provide an overview of the currently known Ichthyopterygia from Switzerland. Their record spans from the Middle Triassic to the Early Cretaceous. Both some of the largest and of the smallest ichthyopterygian taxa occur in the Swiss Triassic. The species *Wimanius odontopalatus, Mixosaurus cornalianus, M. kuhnschnyderi, Cymbospondylus buchseri*, and *Argovisaurus martafernandezi* are based on Swiss type specimens. It is remarkable that not all of the materials are from conservation deposits. At least 13 species have become known from the Swiss Mesozoic and are shortly portrayed here. Especially the exceptionally preserved skeletons from Monte San Giorgio, as well as the partial skeleton of the Jurassic *Argovisaurus* provided important details to improve our understanding of the evolution of the clade.

## Data Availability

The repository of the mentioned specimens is indicated including specimen numbers. All figured specimens are available for study in public collections. All data included in our analyses have been published previously and the sources are provided in Tables 1, 2, 3.
